# Non-ionotropic voltage-gated calcium channel signaling

**DOI:** 10.1080/19336950.2024.2341077

**Published:** 2024-04-11

**Authors:** Michael Trus, Daphne Atlas

**Affiliations:** Department of Biological Chemistry, Institute of Life Sciences, The Hebrew University of Jerusalem, Jerusalem, Israel

**Keywords:** Excitation transcription (ET) coupling, CICR, cardiac excitation contraction (EC) coupling, synaptic transmission, L-type Ca^2+^ channel, conformational coupling

## Abstract

Voltage-gated calcium channels (VGCCs) are the major conduits for calcium ions (Ca^2+^) within excitable cells. Recent studies have highlighted the non-ionotropic functionality of VGCCs, revealing their capacity to activate intracellular pathways independently of ion flow. This non-ionotropic signaling mode plays a pivotal role in excitation-coupling processes, including gene transcription through excitation-transcription (ET), synaptic transmission via excitation-secretion (ES), and cardiac contraction through excitation-contraction (EC). However, it is noteworthy that these excitation-coupling processes require extracellular calcium (Ca^2+^) and Ca^2+^ occupancy of the channel ion pore. Analogous to the “non-canonical” characterization of the non-ionotropic signaling exhibited by the N-methyl-D-aspartate receptor (NMDA), which requires extracellular Ca^2+^ without the influx of ions, VGCC activation requires depolarization-triggered conformational change(s) concomitant with Ca^2+^ binding to the open channel. Here, we discuss the contributions of VGCCs to ES, ET, and EC coupling as Ca^2+^ binding macromolecules that transduces external stimuli to intracellular input prior to elevating intracellular Ca^2+^. We emphasize the recognition of calcium ion occupancy within the open ion-pore and its contribution to the excitation coupling processes that precede the influx of calcium. The non-ionotropic activation of VGCCs, triggered by the upstroke of an action potential, provides a conceptual framework to elucidate the mechanistic aspects underlying the microseconds nature of synaptic transmission, cardiac contractility, and the rapid induction of first-wave genes.

## Introduction

Voltage-gated calcium channels (VGCCs) play a pivotal role in mediating excitation-coupling processes including excitation secretion (ES) coupling, excitation transcription (ET) coupling, as well as skeletal and cardiac contraction excitation contraction (EC) coupling.

The main focus in investigations of ES, EC, and ET coupling has been centered on the L-type calcium channels (Cav1.2; Cav1.3), which are abundantly expressed in neuronal, neuroendocrine, and non-neuronal cells [1].

Traditionally, the primary function of VGCCs has been focused on calcium (Ca^2+^) entry, culminating in the subsequent activation of Ca^2+^-dependent intracellular signaling pathways. However, this classic view characterizing VGCCs solely as ion-conducting pores destined to bring calcium ions into the cell falls short in elucidating microsecond-scale excitation-coupled processes, such as action-potential-triggered vesicle fusion events.

A direct functional interface between VGCCs and transmembrane-spanning exocytotic proteins has been identified [2–8]. These findings strongly indicated the presence of a potential conformational-triggered signaling mechanism operating at microsecond timescales. The kinetics of synaptic vesicle fusion events initiated during excitation-secretion coupling revealed a short latency period of approximately 60 µs between the initiation of presynaptic membrane depolarization and the subsequent postsynaptic response [9]. This rapid fusion event may be facilitated by the functional and physical interplay between voltage-gated calcium channels (VGCCs) and exocytotic proteins (reviews [10–12]).

Further studies have demonstrated that synaptic vesicle fusion, which is triggered during membrane depolarization is mediated through the Ca^2+^-impermeable L-type channel (Cav1.2^L745P^). This findings has given rise to the propositions of a non-ionotropic, conformational-triggered signaling process [13–15].

Although Ca^2+^ entry is not necessary, synaptic transmission mediated by non-ionotropic conformational coupling remains critically dependent on extracellular Ca^2+^, thereby underscoring the essential role of Ca^2+^ binding to the channel ion pore [13–16].

Experimental evidence has provided additional support for the hypothesis of conformational signaling that operates independently of Ca^2+^ influx, yet necessitates extracellular cations for ion-pore occupancy. This was reinforced by demonstrating vesicle fusion triggered by substituting Ca^2+^ with impermeable lanthanum cations, which are known to occupy the ion pore of the channel [14–17].

In accordance with the proposed non-ionotropic model, the VGCC acts as a macromolecule that triggers intracellular activity through trans-membrane-spanning intracellular proteins, akin to non-ionotropic N-methyl-D-aspartate receptor (NMDAR) signaling.

Interestingly, the skeletal-muscle contraction coupling (EC) has long been known to be triggered independently of Ca^2+^-entry. This occurs through a direct interaction between the channel Cav1.1 and the intracellular protein ryanodine receptor1 (RyR1) [18–20].

The facilitation of cardiomyocyte contraction by the Ca^2+^-impermeable Cav1.2 channel led to the proposal of non-ionotropic cardiac muscle excitation-contraction (EC) coupling [21]. This delineation of a non-ionotropic pathway of cardiac contraction diverges from the conventional ionotropic mechanism of calcium-induced calcium release (CICR).

Recent reports have revealed that similar to ES coupling, excitation transcriptional (ET) activation is mediated by Cav1.2 in a non-ionotropic manner, as demonstrated in studies conducted by Servili et al. [22–24]. This activation, initiated by a depolarizing signal during ion-pore occupancy and preceding Ca^2+^ inflow, is consistent with earlier report indicating that dendritic growth and arborization are facilitated by non-ionotropic signaling [25].

Taken together, these studies provide substantial evidence for an ion flux-independent molecular signaling mechanism that drives excitation-coupled processes in response to membrane depolarization. Activity mediated by VGCC encompasses two categories: non-ionotropic activity characterized by Ca^2+^ occupancy of the ion pore independent of Ca^2+^ inflow, and ionotropic activity associated with ion influx and subsequent elevation of intracellular calcium.

In this review, we discuss these findings and their implications to increase our understanding of the mechanism by which VGCCs mediate gene transcription, microsecond synaptic transmission, and cardiac contractility.

## The voltage-gated calcium channel

### The voltage-gated calcium channel

Voltage-gated calcium channels (VGCCs) are multi-subunit complexes comprising a pore-forming α_1_ subunit and three auxiliary independent subunits, β, α2δ, and γ. The α_1_ subunit is a single polypeptide consisting of 24 transmembrane segments, separated by intracellular and extracellular links. The Ca^2+^ pore is formed in the center of four homologous domains (I-IV) consisting of six transmembrane segments each. The S1–S4 segments of each domain are voltage sensors located at the periphery of the pore, and segments S5 and S6 are connected via the P-loop forming the central pore itself. The β subunit is an intracellular subunit, α2δ is an extracellular membrane-associated disulfide-linked subunit, and γ is a transmembrane subunit. The auxiliary subunits β and α2δ, participate in α_1_ trafficking to the cell membrane and regulate the activation and inactivation kinetics of the channel. For more details, refer to recent reviews [1,26–28].

Voltage-dependent activation is initiated by the outward movement of the positive gating charges, which open the ion-pore within the α_1_ subunit to allow Ca^2+^ binding preceding Ca^2+^ conductance into the cell. Cation binding occurs at a specific site within the pore, formed by four glutamate residues, called the EEEE motif, which determines the cation affinity, selectivity, and conductivity.

The detailed conformational changes and distribution of Ca^2+^ sites within the channel selectivity filter, EEEE motif, and mechanism of ion conductance are not fully understood [29]. In addition, the presence and contribution of the blocking lipid polar headgroup, which is in direct coordination with the Ca^2+^ ions within the selectivity filter, is not fully understood [30].

A tentative configuration based on experimental and theoretical evidence suggests that in the closed state, the EEEE motif of α1.2 subunit of Cav1.2 is occupied by a single strongly bound calcium ion (Kd ~1 µM). It is assumed that the conformational change(s) that open the channel during an action potential (AP) generates a low-affinity multiple calcium ion-binding site (Kd = 13.6 mM), which enables picoampere currents of ~10^9^ ions/s [31].

## Non-ionotropic excitation transcription (ET) coupling

### The prevailing model

VGCC-dependent synapse-to-nucleus communication, or ET coupling, has been shown to regulate multiple cellular activities, including cell proliferation, differentiation, and transcription of immediate-early genes such as c-*fos*, and activation of synaptic molecules such as nNOS, Bcl-2, and brain-derived neurotrophic factor (BDNF) [32–34]. The activity and genetic variants of Cav1.2 subunits, including the pore-forming α_1_1.2 and the auxiliary β subunit, were shown to be associated with the transcription of downstream factors such as schizophrenia, attention deficit hyperactivity and autism associated disorders (ASD).

In early studies, depolarization-triggered transcription was reported by monitoring the phosphorylation of cyclic adenosine monophosphate (cAMP) response element-binding protein (CREB) at Ser^33^ [35]. The involvement of CREB in this context highlights its role as a key transcription factor that responds to changes in cellular membrane potential. The increase in cytoplasmic calcium [Ca^2+^]_i_ following the opening of Cav1.2, has been proposed to facilitate the phosphorylation of CREB through the activation of several pathways, including the Ca^2+^/calmodulin (CaM)-dependent kinase IV (CaMKIV), Ca^2+^/CaM-dependent kinase II (CaMKII), adenylate cyclase/cAMP/PKA, and the Ras/MAP kinase (MAPK) pathways [36–40].

For example, transcriptional activity in rat superior-cervical ganglion neurons triggered during membrane depolarization has been attributed to an elevation of [Ca^2+^]_i._ and subsequent binding to CaMKII, characterized as a weakly sensitive local Ca^2+^ sensor associated with CaMK activation [41].

Additional studies have proposed a mechanism by which a shuttle that transports Ca^2+^/calmodulin from the surface membrane to the nucleus is activated through Ca^2+^ binding to both βCaMKII and calcineurin (CaN) [42,43]. Accordingly, the binding of γCaMKII to calcium/CaM near the membrane and subsequently the phosphorylation of α/βCaMKII, traps the calcium/CaM cargo. Dephosphorylation by CaN directs the Ca^2+^/CaM-bound kinase to the nucleus by exposing the nuclear localization signal. In the nucleus CaMKK activation by Ca^2+^/CaM further activates the CREB kinase signaling [42–44]. Interestingly, in these studies, ET coupling was shown to be highly sensitive to channel open probability and less sensitive to changes in [Ca^2+^]_i_ [41–43].

In vascular myocytes ET coupling has been shown to be mediated by a molecular complex comprising of Cav1.2/CaMKK2/CaMK1a localized to caveolae converting [Ca^2+^]_i_ changes into gene transcription [45].

Overall, the predominant paradigm posits that elevation in [Ca^2+^]_i_ is responsible for gene activation, whether conducted subsequent to Ca^2+^ influx (*I*_ca_) through Cav1.2, NMDARs, or Ca^2+^ release from the endoplasmic reticulum via ryanodine or IP_3_ receptors, and conceivably, the nuclear envelope [46].

### Non-ionotropic ET coupling, the alternative model

An early study showed that Cav1.2 mediates dendrite retraction via RhoA signaling independently of Ca^2+^ inflow [25]. This study introduces a novel perspective on calcium-influx independent ET coupling, aligned with studies of non-ionotropic transcriptional activation triggered by the NMDA receptor (NMDAR) [47–55].

More recently, several studies have investigated non-ionotropic Cav1.2 mediated gene activation in PC12 cells, SH-SY5Y cells, and in HEK293 cells co-expressing α_1_1.2, β_2b_, and α2δ Cav1.2 subunits.

In these cells, membrane depolarization (70 mM KCl for 3 min) resulted in an increase in the phosphorylation of ERK1/2, RSK, and CREB, as well as in an increase in c-Fos, c-Jun, and MECP2 expression [22–24,56], consistent with signaling in the nucleus by Cav1.2 [36].

In HEK293 cells co-expressing Cav1.2 subunits, ET coupling was abolished by omitting the β subunit, or by introducing a specific mutation within the alpha-interacting-domain (AID) at α_1_1.2, which disrupts the high affinity interaction site between the Cav1.2 α_1_ and β subunits [23]. These results implicate the β subunit in the control of gene expression, highlighting the integral participation of the β subunit in the activation of H-Ras, upstream of the Raf/MEK/ERK/RSK and CREB pathway.

This molecular pathway of ET coupling was substantiated through pulldown studies which revealed a direct physical interaction between Cavβ_2b_ or Cavβ_2a_ and the Ras exchangers RasGRF1 or RasGRF2. Accordingly, a depolarizing signal is transmitted from α_1_1.2 to the β subunit, mediating ET coupling, subsequent to H-Ras activation by RasGRF2 [23,56].

In an earlier study, a functional and physical interaction between Cav1.3 and RyR2 was shown to be responsible for translating channel activity into gene activation [57].

Non-ionotropic-mediated gene activation was investigated in HEK293 cells co-expressing the non-conductive α1.2^L745P^ subunit along with β2a and α2δ. The L745P mutation in the human α1.2 subunit corresponds to the Ca^2+^-impermeable α1.2^L775P^ rat mutant [58]. Membrane depolarization of cells expressing the impermeable channel mutant triggered activation of the Ras/ERK/CREB pathway, resulting in an increase in the expression of the transcriptional factors MECP2, c-Fos, and c-Jun, consistent with a non-ionotropic mechanism of ET coupling [22,23].

Further corroboration of ET coupling mediated via the Ras/ERK/CREB nuclear signaling pathway has been demonstrated by the Cav1.2 missense variant commonly associated with the neurodevelopmental disorder Timothy Syndrome [59]. The single point Cav1.2 mutant G406R at the α_1_1.2 subunit, primarily affects the heart and is associated with autism-related disorders attributed to a constitutive gene activation [59].

Comparable to wt Cav1.2 and the Ca^2+^-impermeable mutant Cav1.2^L745P^, both the Timothy mutant (Cav1.2^G406R^) and the Ca^2+^-impermeable Timothy mutant (α_1_1.2^G406R/L745P^) mediate transcriptional activation via the Ras/ERK/CREB pathway [24,56,60].

The Ca^2+^-inflow independent transcription mediated via the Ras/ERK/RSK/CREB pathway appears to be critically dependent on conformational changes transmitted during membrane depolarization from the α_1_1.2 to the β subunit of the VGCC [23,24].

These results demonstrate that during membrane depolarization Cav1.2 can initiate intracellular signaling independent of Ca^2+^ influx, similar to a non-inotropic signaling mediated by NMDA receptors [47–55].

Finally, transcriptional coupling triggered independently of Ca^2+^ entry has also been demonstrated by comparing activation by the two Timothy mutants, G406R and the G402S [24]. Although both mutants induce an increase in Ca^2+^ entry and compromise voltage-dependent inactivation (VDI), only G406R is linked to behavioral and autistic disorders [61]. The hyperpolarizing shift in voltage-dependent activation kinetics observed in the G406R mutant, but not in the G402S mutant, appears to be correlated with spontaneous gene activation. Constitutive activation of the Timothy channel Cav1.2^G406R^ predicted by the hyperpolarizing shift, has been suggested to be responsible for autistic and behavioral activities [24,56].

### ET coupling requires Cav1.2 ion-pore occupancy

To establish the pivotal role of Ca^2+^-occupancy within the ion-pore in ET coupling preceding the onset of Ca^2+^ influx, Servili et al., employed selective ion-pore mutants of the calcium impermeable channel Cav1.2^L745P^ [23].

**Figure 1.**
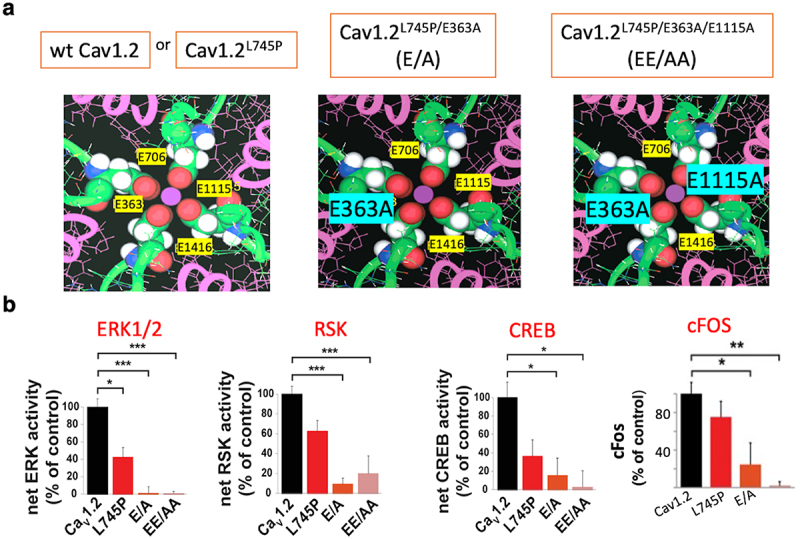
Excitation transcription coupling is critically dependent on Ca^2+^ binding to the selectivity filter. (a) A proposed structural model of the EEEE motif, four glutamate comprising high-affinity Ca^2+^ binding site of the channel ion pore, according to Lipkind and Fozzard [62] (*upper, left*). A single point selectivity filter mutant of the Ca^2+^-impermeable channel;, α_1_1.2^L745P/E363A^ (*upper, middle*), and a double point selectivity filter mutant of the Ca^2+^-impermeable channel, α_1_1.2^L745P/E363A/E1115^ mutant (*upper, right*) (b) HEK293 cells expressing the wt, impermeable mutant or the pore mutants stimulated by 70 mM KCl for 3 min. Phosphorylation of ERK1/2, RSK, and CREB monitored after stimulation and c-Fos expression, 60 min later, monitored by western blot analysis. Quantification plotted as averages (±SEM) of 3 independent experiments normalized to the corresponding non-phosphorylated proteins using the corresponding antibodies. All experiments were done in triplicate transfections and performed 3 times using different cell batches. One-way ANOVA was performed to determine statistically significant differences for K70-stimulated cells. *p < 0.05, **p < 0.01, ***p < 0.001. Adapted from [23].

The single-point mutant E363A (E/A) and the double-point mutant E363A/E1115A (EE/AA) were each introduced into the EEEE motif of the Ca^2+^-impermeable channel Cav1.2^L745P^. ET coupling triggered in HEK293 cells expressing the single ion pore mutant α_1_1.2^L745P/E363A^/β2b/α2δ (EA) exhibited a significant reduction in the phosphorylation of ERK1/2, RSK, CREB, and c-Fos expression (>80%), while ERK1/2, RSK, and CREB phosphorylation or gene expression were virtually undetectable by the double pore mutant α_1_1.2^L745P/E363A/E1115A^/β2b/α2δ (EE/AA) (Figure 1). A compromised ET coupling by channel pore mutants supports a key role of a Ca^2+^-occupied selectivity filter during channel opening.

#### ET coupling by ion-pore impermeable La^3+^

The importance of ion-pore occupancy in ET coupling has been further demonstrated through the substitution of Ca^2+^ with La^3+^, an ion pore impermeable cation. La^3+^ has been shown to support depolarization-triggered catecholamine release in bovine chromaffin cells [15,16,63], and insulin release from insulinoma cells [17]. Upon substituting Ca^2+^ with La^3+^ both wt Cav1.2 and Timothy channel Cav1.2^G406R^ exhibited activation of the Ras/ERK/CREB pathway. These results are consistent with conformational-coupling signaling mediated by the non-ionotropic activity of the channel [22,24].

### Involvement of [Ca^2+^]_i_ and Ca^2+^/CaM in ET coupling

The molecular mechanism underlying calcium-dependent inactivation of the channel involves Ca^2+^/calmodulin (CaM) binding to the C-terminal IQ domain of Cav1.2. A mutation introduced in the IQ domain of α_1_1.2 at I1624A, designed to prevent Ca^2+^/CaM binding, resulted in only a minor reduction in depolarization-induced ERK1/2 phosphorylation and showed no effect on the activation of RSK or CREB, when compared to wt α_1_1.2. These findings indicate a lack of significant involvement of [Ca^2+^]_i_ in the transcriptional activity mediated via the Ras/ERK/CREB pathway [23].

**Figure 2.**
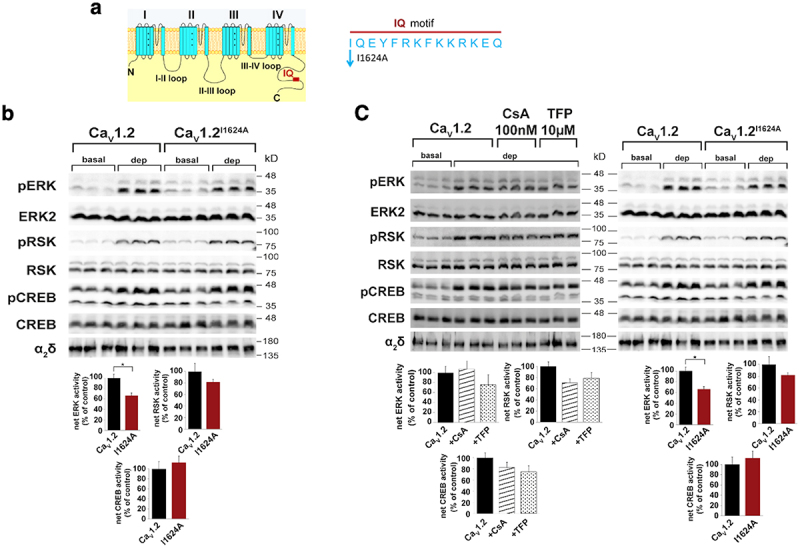
The IQ motif of Cav1.2, Ca^2+^/CaM, or calcineurin, are not involved in ET coupling. (a) Schematic view of the IQ calmodulin (CaM)-binding motif location at the C-tail of the α_1_1.2 subunit. (b) *The contribution of IQ motif to ET coupling* HEK293 cells transfected with wt Cav1.2 (α_1_1.2/β2b/α2δ) or the IQ Cav1.2 mutant (α_1_1.2^I1624A^/β2b/α2δ) treated 72 hr later with non-depolarizing (2.5 mM KCl; basal) or depolarizing (70 mM KCl; dep) solutions for 3 min. Phosphorylation of ERK, RSK, and CREB was detected using the corresponding anti-phospho-protein antibodies (*upper*) and quantified normalizing with antibodies of the corresponding non-phosphorylated proteins and anti α2δ subunit antibodies (*lower)*. (c) *The effect of cyclosporine a (CsA) the selective CaN inhibitor, and trifluoperazine (TFP), a Ca*^*2+*^*/CaM inhibitor, on ET coupling* HEK293 cells transfected with wt Cav1.2 (α_1_1.2/β2b/α2δ) or the IQ mutant (α_1_1.2^I1624A^/β2b/α2δ). Seventy-two hr after transfection the cells were treated with 100 nM CsA or 10 μM TFP for 2 hr, the cells were pulsed with 2.5 mM KCl; basal, or 70 mM KCl; dep, solutions for 3 min. One-way analysis of variance (ANOVA) was used to determine statistically significant differences. The plotted values of net phosphorylation are averages (±SEM) of three independent experiments normalized to the corresponding non-phosphorylated proteins. Adapted from [23].

Furthermore, the Ras/ERK/CREB pathway was not affected either by trifluoperazine, a Ca^2+^/CaM inhibitor, or by cyclosporine A (CsA), a selective inhibitor of Ca^2+^-dependent protein phosphatase (CaN) (Figure 2) [23]. It is well established that CaN accelerates inactivation of Cav1.2 inward-currents during depolarization acting in a Ca^2+^/CaM-dependent manner [64].

Although ET coupling in channel transfected HEK293 cells appears to be mediated exclusively via the Ras/ERK/CREB pathway, one cannot exclude the possibility that ET coupling in other cells can also be triggered via Ca^2+^/CaM activation.

In PC12 cells, similar to Cav1.2 transfected HEK293 cells, ET coupling upregulated MECP2, BDNF, c-Fos, and c-Jun expression through the Ras/ERK/CREB pathway [22]. Moreover, it has been demonstrated that ET coupling can be initiated even when extracellular Ca^2+^ is substituted with Ba^2+^ at the extracellular medium. Since Ba^2+^ ions bind to the selectivity filter of Cav1.2 and do not bind to calmodulin, transcription in these cells with Ba^2+^ reinforces the significance of ion-pore occupancy in triggering conformational ET coupling, as opposed to ion influx.

Transcriptional programming studies have proposed that varying neuronal activity patterns can induce different sets of activity-regulated, primary-response-genes (PRGs) and secondary response genes (SRGs) [65]. Two kinetically distinct classes of PRGs have been identified in neurons, rapid PRGs (rPRGs) and delayed PRGs (dPRGs). The activation of rapid rPRGs, also known as immediate early genes [IEGs], is mechanistically distinct from the later sustained gene program of dPRG or *de novo* translation-dependent secondary response gene transcription [65–67]. According to this study, most of these rapid rPRGs, which represent the early stages of gene induction, appear to be induced via the ERK/MAPK pathway [67]. ERK/MAPK inhibitors inhibit rapid rPRGs and exhibit a minimal impact on the expression of dPRG in membrane-depolarized neurons.

**Figure 3.**
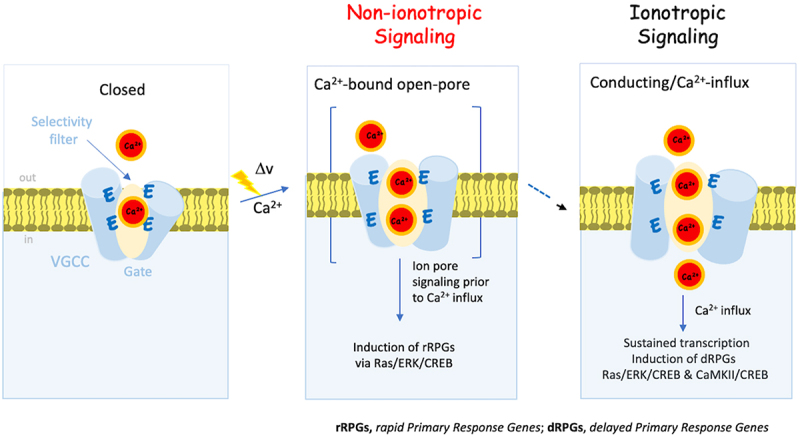
The Non-ionotropic activity of Cav1.2 mediates excitation transcription (ET) coupling. A schematic illustration of VGCC in the closed state, occupied with a single Ca^2+^ ion tightly bound (<1 µM) to the EEEE motif (*left)*. Upon arrival of an action potential (XXXv) the open channel now occupied with an additional Ca^2+^ ion triggers transcription of immediate early genes (IEGs), also known as Primary-Response-Genes (rPrgs), prior to and independent of ion flow (non-ionotropic activity) (*middle*). Subsequent Ca^2+^ influx (ionotropic activity) and elevation of [Ca^2+^]_i_ might suggest transcription of delayed Primary-Response-Genes (dPrgs), either via the CaM-Ca^2+^ dependent pathway and/or other intracellular Ca^2+^ dependent processes (*right*).

In accordance with the findings of these studies, it is tempting to speculate that transcription of rPRGs occurs during channel opening and ion-pore occupancy, prior to and independent of Ca^2+^-entry. In contrast, the transcription of dPRG and SRGs may necessitate prolonged stimulation and a more substantial increase in intracellular calcium concentration ([Ca^2+^]_i_) (Figure 3). This speculative interpretation involving gene transcription in a rapid Ca^2+^ influx-independent process and a slower Ca^2+^ entry-dependent process in response to varying patterns of neuronal activity and Ca^2+^-signaling dynamics requires further research.

#### In summary,

The evidence presented indicates that excitation-contraction (ET) coupling can be mediated by Ca^2+^-impermeable channels or by substituting Ca^2+^ with the ion-pore impermeable cation, La^3+^. This Ca^2+^ inflow-independent signaling reinforces the conceptual model of a non-ionotropic mechanism, suggesting conformational coupling between VGCC and gene expression.

In the proposed model, the transmission of a signal initiated by an action potential is conveyed from the voltage-sensing α_1_1.2 subunit of Cav1.2 to the β subunit, triggering transcription through sequential protein–protein interactions. This conformationally triggered signaling is mediated via the Ras/ERK/CREB pathway. Transcription is critically dependent on ion-pore occupancy and on the β subunit, which physically interacts with the H-Ras exchangers RasGRF1 and RasGRF2.

One may speculate that transcription of rapid primary response genes (rPRGs) is triggered by non-ionotropic conformationally coupled signaling. On the other hand, transcription of the slower wave of delayed primary response genes (dPRGs) or secondary response genes (SRGs), which rely on protein induction and requires prolonged stimulation, and most likely, a higher elevation of intracellular calcium concentration, can be induced through Cav1.2 ionotropic signaling, involving Ca^2+^ binding to Ca^2+^/CaM and activation of CaMKII.

The hypothetical coexistence of Ca^2+^ influx independent and Ca^2+^ influx dependent ET coupling, associated with the activation of distinct gene programs, is illustrated in Figure 3. This implies graded and selective induction of rapid immediate-early genes (IEGs) followed by a Ca^2+^-influx-dependent and sustained gene activation process.

## Non-ionotropic excitation secretion (ES) coupling

### The Prevailing model, a Ca^2+^ influx dependent evoked synaptic transmission

Voltage-gated calcium channels (VGCCs) comprising of Cav1.2, and Cav1.3 (L-type), Cav2.1 (N-type), Cav2.2 (P/Q-type), and Cav2.3 (R-type) play a crucial role in depolarization-evoked transmitter release. The channels transduce an electrical signal and trigger vesicle fusion in a process called excitation-secretion (ES) coupling. This coupling process is dependent on the presence of extracellular Ca^2+^ and takes place within the active zones of synapses during the upstroke of an AP [1,26]].

The temporal coordination between the opening of the VGCCs, Ca^2+^ entry, and vesicle fusion during ES coupling was remarkably rapid. Studies such as that by Sabatini and Regehr in 1996 have indicated that VGCC-driven vesicle fusion lags behind the opening of the channels by approximately 60 microseconds [9]. It is now widely accepted that “it takes as little as 100 microseconds from the arrival of an action potential to the release of neurotransmitters by Ca^2+^-evoked synaptic vesicle exocytosis” [68].

The requirement for extracellular Ca^2+^ led to the seemingly evident perspective that Ca^2+^ influx and the subsequent elevation of [Ca^2+^]_i_ are essential for ES coupling.

The current prevailing model posits that SNARE proteins, specifically syntaxin 1A (Sx1A), synaptosomal-associated-protein, 25 kDa (SNAP-25), and synaptobrevin, assemble into a compact complex. This arrangement facilitates close apposition of the vesicle with the plasma membrane, which is crucial for vesicle fusion. The subsequent disassembly of these SNARE complexes according to this model, is mediated by soluble NSF attachment proteins (SNAPs) and N-ethylmaleimide-sensitive factor (NSF). This disassembly activates several regulated cellular steps initiated by Munc18–1 binding to the “closed” conformation of Sx1A. Through binding to synaptobrevin Munc18–1 bridges the vesicle with the plasma membrane, assembling the SNARE complex during of Sx1A “opening” by Munc13–1. Vesicle fusion is prevented when vesicular synaptotagmin (Syt1) and complexin bind to partially assembled SNARE complexes. Fusion occurs upon Ca^2+^-binding to Syt1, leading to dissociation from the SNARE complex. Hence, subsequent to Ca^2+^ binding to the C2 domains of Syt1, the synaptic vesicle fuses with the plasma membrane through zippering of the SNARE complex and unlocking a Syt1/SNAREs/complexin complex [69–76].

In this prevailing model, Ca^2+^ binding to Syt1 is simultaneously involved in two major consecutive Ca^2+^ binding steps: vesicle priming and vesicle fusion. However, the details of these processes that occur upon Ca^2+^ binding to Syt1 are yet to be clarified.” [77].

A total-internal-reflection-fluorescence (TIRF) study of cerebellar mossy fiber (cMF) terminals enabled to discrimination between Ca^2+^-priming and Ca^2+^-mediated fusion steps, revealing a Ca^2+^-dependent vesicle priming phase, coupled with a fast Ca^2+^-dependent vesicle fusion event [78]. This study implies the potential involvement of an additional Ca^2+^-binding protein in the initiation of µsec Ca^2+^-dependent vesicle fusion [11]; (further elaboration on this aspect is presented in the subsequent section).

### A non-ionotropic alternative model

#### Functional interaction of Cav1.2, Cav2.1, Cav2.2, and Cav2.3 with synaptic proteins

As reviewed above, the prevailing model does not incorporate VGCCs as active players in the fusion event, despite their functional and physical interactions with exocytotic proteins and their recognized position within the AZ.

To elucidate the role of VGCCs and Ca^2+^ in mediating µsec fusion events, an alternative model was proposed. It incorporates VGCCs alongside the exocytotic proteins, and is aligned with the Ca^2+^ nanodomain model of evoked-release.

Initial biochemical studies combined with voltage-clamp recordings of microinjected *Xenopus* oocytes and patch-clamped single-cell release measurements in pancreatic β-cells revealed functional complexes assembled through physical interactions of VGCC with Sx1A, SNAP25, and Syt1 [2,5–7,11,79–83]. Voltage-clamp recordings have shown that the kinetics of activation, inactivation, and steady-state inactivation of Cav1.2, Cav2.1, Cav2.2 and Cav2.3 are modulated by the SNARE proteins Sx1A, SNAP-25, or Sx1A/SNAP-25 combined [3,5–7,82,84,85].

Extensive biochemical studies have identified bidirectional communication between Sx1A and SNAP-25 with the cytosolic loop that separates segments II-III of the α_1_1.2 subunit (Cav1.2 II-III_753–893_ loop) or a shorter sequence at α_1_2.1 subunit (Cav2.1 II-III_773–859_ (*synprint)*) with Sx1A_181–288_ [4–7,82,86].

**Figure 4.**
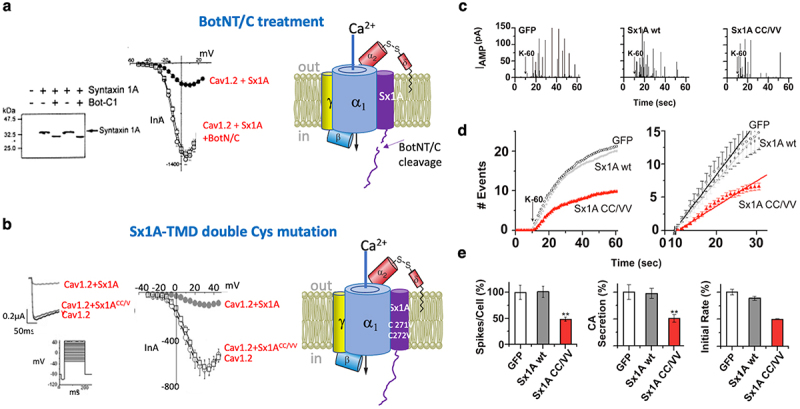
Intra-membrane signaling between the voltage-gated Ca^2+^ channel and syntaxin 1A coordinates synchronous release. (a) *Botulinum C1 (BotNT/C1) cancels the effect of syntaxin 1A (Sx1A) on Cav1.2 current amplitude*. Expression of Sx1A with Cav1.2 with or without BotNT/C light chain in *Xenopus* oocytes injected with cRNA of α_1_1.2/β2A/α2δ subunits. At day 2 after injection with the channel subunits, oocytes were injected with cRNA Sx1A, and at day 4 with cRNA BotNT/C light-chain. Protein expression at day 6 was monitored by western blot with anti Sx1A antibodies(*left)*. Ca^2+^ currents were evoked from a holding potential of −80 mV by a single voltage step of 140 ms duration to a step potential of +20 mV. Depolarization triggered leak-subtracted peak current-voltage relationship (see collected data from oocytes expressing α_1_1.2/β2a/α2δ subunits alone (open circle), with Sx1A (closed circle), with Sx1A and BotNT/C (square) (*middle)*; (Student’s *t*-test, *p* < 0.01 (*n* = 6–9). Adapted from ref [83]. Schematics of Cav1.2 in a complex with BotNT/C cleaved Sx1A *(right*); plotted to concur with literature reports, for example, refs [83,88]. (b) *Mutating Cys-271 and Cys-272 of the Sx1A TMD disrupts Sx1A interaction with Cav1.2*. Oocytes were injected with α_1_1.2/α_2_δ/β2a and at day 2 with Sx1A or Sx1A double mutant Sx1A^CC/VV^. at day 6 after injection, Ca^2+^ currents were evoked from a holding potential of −80 mV by a single voltage step of 140 ms duration to a step potential of +20 mV in oocytes expressing the three channel subunit, with and without Sx1A or Sx1A^CC/VV^ (*left*). Leak-subtracted peak current-voltage relationship (see *inset)*: collected data from oocytes expressing the three channel subunits (open circle) together with Sx1A (closed circle) or Sx1A^CC/VV^ (open rectangle) *(middle)*.The data points correspond to the mean ± S.E. of current amplitude (*n* = 8). Adapted from [89]. Schematic presentation of Cav1.2 in a complex with the Sx1A TMD mutant (Sx1A^CC/VV^), (*right*); plotted to concur with literature reports [90]. (c) *Dominant inhibitory effect of Sx1A*^*CC/VV*^ *transmembrane mutant on depolarization-evoked catecholamine release in bovine chromaffin cells* Amperometry traces for control, GFP-infected, RFP-wt Sx1A infected, and RFP-Sx1A^CC/VV^ infected cells elicited by a pulse of 60 mM KCl (K60), indicated by the arrow. (d) Cumulative spike counts plotted versus time illustrate the time course of catecholamine secretion triggered by membrane depolarization (starting at *t* = 10; *Left*). Expanded view of the initial cumulative spike counts shown in (c) (*right)*. (e) Average number of spikes elicited per cell (*Left*). The total mean charge represents total CA secretion, which is the average area underneath the spikes and is presented as the percentage of average secretion per cell *(Middle)*, the mean frequency of the initial rate was calculated as the maximum slope in plot (b) (*right*) during the first 30 sec of recording **p < 0.005 Adapted from ref [79].

Evoked-insulin secretion in single pancreatic β-cells injected with the recombinant intracellular loop Cav1.2 II-III_753–893_ confirmed a functional coupling between the cytosolic domains of the channel and SNAREs, consistent with channel/synaptic-protein interactions rather than Ca^2+^ entry [7,80,83]. A regulatory association between the channel and Sx1A was also confirmed by the negative-impact of Sx1A on current amplitude in oocytes expressing Cav1.2 or Cav2.1 [24,81,83,84,87] (Figure 4(a)). The reversal of Sx1A inhibition by Botulinum Neurotoxin type C (BotNT/C), which cleaves Sx1A at a single amino acid and blocks transmitter release [91], provides yet an additional evidence for the involvement of the Sx1A/channel interdependence interaction in depolarization-evoked release [81,83,84] (Figure 4(a)).

In addition to cytosolic protein-protein interactions, a transmembrane interface between Sx1A and the channel was identified. This interface was detected through the reversal of Sx1A effects on channel kinetics, achieved by mutations at the highly conserved vicinal residues Cys271 and Cys272 within the transmembrane domain (TMD) of Sx1A [81,83,89,92] (Figure 4(b)). Dominant negative recordings of either the single Sx1A^C271V^, Sx1A^C272V^, or the double point Sx1A^C271V/C272V^ TMD mutants significantly inhibited depolarization-triggered catecholamine release (CA) release in single chromaffin cells [79,89] (Figure 4(c-e)). The reconstituted evoked-release, as monitored by capacitance recordings in *Xenopus* oocytes co-expressing Cav1.2 or Cav2.2, along with Sx1A/SNAP-25/Syt1, was significantly disrupted by the expression of a recombinant intracellular domain Cav1.2 II-III_753–893_. Additionally, mutations at the polylysine motif in the C2A domain of Syt1, a recognized interaction site with the channel, resulted in elimination of depolarization-evoked capacitance transients, consistent with protein–protein crosstalk between the Ca^2+^ channel and SNAREs [88,93].

### The redox sensitivity of depolarization evoked-release

**Figure 5.**
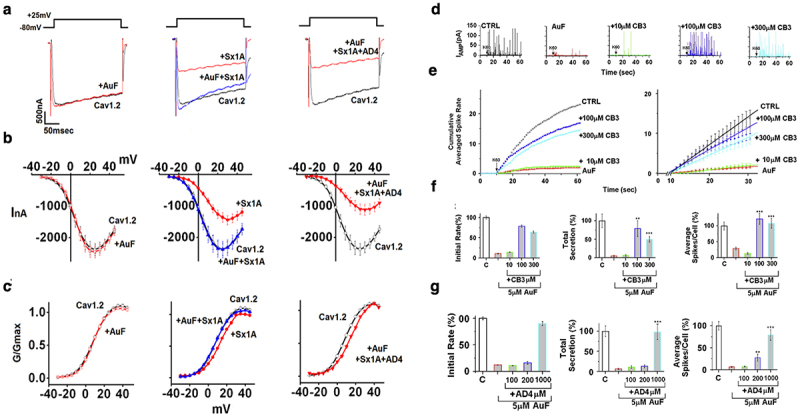
Oxidation of Sx1A disrupts the α_1_1.2/Sx1A intracellular signaling and blocks evoked-transmitter release. (a) Representative superimposed α_1_1.2/β2/α2δ current traces (Cav1.2) in the absence and in the presence of AuF (*left*); Sx1A/Sx1A + AuF as indicated *(middle)* or Sx1A+AuF+AD4 *(right)*. (b) Leak subtracted current-voltage relationships of α_1_1.2/β2/α2δ expressed either alone (open circles) or with AuF (open rectangle *left*); Sx1A/Sx1A + AuF (●/,^▴^ middle) or Sx1A+AuF+AD4 (^◂^, *right*). Inward Ca^2+^-currents evoked from a holding potential of −80 mV to various test potentials in response to 200 ms test pulse at 5 mV increments. (c) G/Gmax values as indicated in (b) The data points correspond to the mean ± SEM of currents (*n* = 8–15). (d) Representative amperometric current traces elicited by a puff of K60 in control cells, AuF-treated cells (5 μM; 30 min), and cells treated with AuF followed by CB3 for 30 min at the indicated concentrations (e) Cumulative events per cell plotted versus time; an expanded view of the initial cumulative spike counts (*right*) and the mean frequency of the initial rate (*left*) (f) Reversal of AuF-induced inhibition of CA release by TXM-CB3. CA secretion elicited by K60 in control cells, AuF-treated cells (5 μM; 30 min), or cells pretreated with AuF followed by TXM-CB3 for 30 min, at the indicated concentrations. The initial rates of CA secretion during the first 20s of recording (*left*), total CA secretion (*middle*), and spikes per cell (*right*) (g) CA secretion elicited by K60 in control cells, AuF-treated cells (5 μM; 30 min), or cells pretreated with AuF followed by NAC-amide (AD4) for 30 min, at the indicated concentrations. The initial rates of CA secretion during the first 20s of recording (*left*), total CA secretion (*middle*), and spikes per cell (*right*). Total secretion calculated as the average picocoulombs at each spike and sustained cumulative spike count. The mean frequency of the initial rate was calculated as the maximum slope during the first 20s of recording. Means were calculated for individual cells as an average of more than 500 spike events adapted from [79,94].

The negative impact of Sx1A on Cav1.2 currents in *Xenopus* oocytes expressing Cav1.2/Sx1A, and on depolarization-triggered catecholamine (CA) release in bovine chromaffin cells is reversibly abolished by application of auranofin (AuF), a thiol-oxidizing reagent [79,94]. The oxidation effect of AuF on Cav1.2/Sx1A currents (Figure 5(a-c)) and on evoked-release in bovine chromaffin cells (Figure 5(d-f)) is fully reversed by thiol reducing reagents such as thioredoxin mimetic peptides like AcCysProCys amide (CB3) (Figure 5(d-f)) or NAC-amide (AD4/NACA) (Figure 5(g)). A significant recovery of CA secretion at 100 μM CB3, was quantified as shown by the cumulative number of events, the maximal slopes, the summation of total secretion, and the number of spikes per cell (Figure 5(d-f)). These results correlate with voltage-clamp *Xenopus* oocyte studies, in which phenylarsine oxide (PAO), a selective Cys vicinal oxidizing thiol reagent, disrupts Sx1A interplay with the channel, and is fully reversed by a reducing reagent [89].

Moreover, super-resolution imaging using two-color photo-activated-localization-microscopy (PALM) revealed nanoscale co-clusters of PAmCherry-tagged Sx1A and Dronpa-tagged α_1_1.2 at a ~ 1:1 ratio in transfected HEK293 cells. This ratio was altered to a ~ 2:1 ratio when the cells were transfected with the functionally inactive transmembrane Cav1.2/Sx1A^C271V/C272V^ mutant instead of wt Sx1A, by oxidation of Sx1A Cys271 or Sx1ACys272 [87]. Thus, a higher ratio level of co-clustering, coincides with compromised depolarization-evoked transmitter-release [87].

Hence, alterations in the channel/Sx1A interface resulting from TMD mutations, oxidation, or BotNT/C cleavage disclose a distinct correlation with the suppression of evoked release. This observation disclosed a direct transmembrane signaling mechanism between the channel and exocytotic machinery, potentially supporting conformation-triggered exocytosis.

Moreover, this mechanism offers insight into the molecular basis of redox sensitivity observed in the release process [12,79,93]. Glucagon exocytosis has been shown to be coupled to Cav2.1 (P/Q-type) channels, similar to the direct coupling of insulin granule exocytosis to the activation of Cav1.2 Ca^2+^ channels [7,95].

### A close proximity between the VGCC and the exocytotic machinery in neurons

A close proximity between the channel and exocytotic machinery that allows for conformational coupling was recently demonstrated in cell-attached recordings from the AZ of Lamprey Reticulospinal Presynaptic Terminals. Performed in acutely dissociated single lamprey giant axon using Lattice Light Sheet microscopy of Ca^2+^ entry, these studies have demonstrated the presence of nanodomains of presynaptic VGCCs coupling with 1:1 stoichiometry with primed vesicles [96].

The total charge entering the axon during 2–3 ms depolarization calculated at each AZ was of 7.98 ± 0.90 fC, which implies a high-affinity Ca^2+^ sensor protein. The authors suggested that Ca^2+^ entry into the AZ through a few open channels, possibly even one, may regulate vesicle fusion. Alternatively, in the context of the non-ionotropic model, an equal number of presynaptic Ca^2+^ channels are localized in close proximity to primed vesicles and form a multi-protein functional complex through protein–protein interactions. In this model, vesicle fusion is driven by conformational coupling mediated by the physical and functional association of VGCCs with synaptic proteins prior to Ca^2+^ influx. These associations have been extensively discussed in previous reviews [10–12,97]. The concept of ES coupling mediated by the non-ionotropic activity of the VGCC determines the precedence in a comparable non-ionotropic signaling mechanism mediated by the NMDA receptors. This emphasizes the ability of ion conducting channels to mediate cellular signaling beyond their classical ionotropic functions [53,55,98,99].

### Ion-pore occupancy mediates transmitter release independent of cation-influx

Two independent experimental approaches were used to investigate and establish non-ionotropic conformation triggered vesicle fusion. First, Ca^2+^ was replaced by a cation that strongly bound to the ion pore of the channel but did not permeate into the cell. Second, a single point mutation is introduced into the channel, resulting in a channel that binds Ca^2+^ without conduction into the cell.

#### Evoked-release mediated by impermeable cation(s) independent of cation-influx

In addition to Ca^2+^, the EEEE motif of Cav1.2 also binds and conducts other divalent cations such as Sr^2+^ and Ba^2+^. Cations from the lanthanide series, including La^3+^, Ce^3+^, and Pr^3+^, which possess ionic radii similar to Ca^2+^, exhibit tight binding to the EEEE motif, but are not conducted intracellularly during membrane depolarization.

Initially, involvement of Cav1.2 occupancy in the exocytotic event was explored by replacing Ca^2+^ with La^3+^, a trivalent cation, recognized for impermeability and high affinity to the Cav1.2 selectivity filter, of ionic radius 1.06Å, similar to Ca^2+^ 1.01Å [16].

Voltage-clamp recordings of oocytes expressing Cav1.2 exhibiting no inward La^3+^, Ce^3+^, or Pr^3+^ currents, confirm trivalent cation impermeability through the selectivity filter [16]. A complementary and highly sensitive (~1 pM) Fura2/La^3+^-fluorescence imaging assay, utilizing La^3+^, further corroborated these findings by demonstrating no buildup of cytosolic La^3+^ and no alterations in cytosolic Ca^2+^ concentrations [15,16].

**Figure 6.**
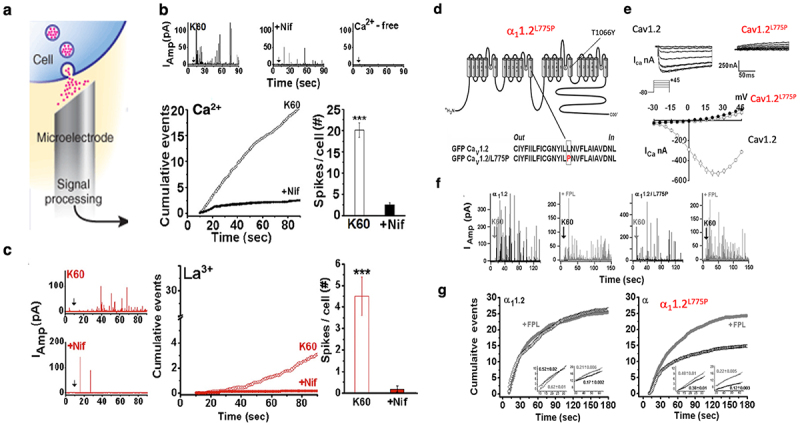
Impermeable cation or calcium-impermeable channel support depolarization evoked secretion. (a) Catecholamine release monitored by amperometry using carbon fiber electrode brought in close proximity of a single bovine chromaffin cell (b) Traces of amperometric currents elicited in bovine chromaffin cells by a 10-s pulse of 60 mM KCl (K60) in the absence and presence of 5 μM nifedipine (Nif) using 2 mM Ca^2+^ as a charge carrier, or in Ca^2+^-free medium (*upper*). Cumulative events per cell plotted versus time (*lower, left*); number of spikes per cell following stimulation shown as mean ± SE; in the presence (*n* = 27) or in the absence (*n* = 36) of 5 μM Nif (*lower right)*. (c) Traces of amperometric currents triggered by K60 in the presence or in the absence of 5 μM Nif, using 0.2 mM La^3+^ in nominally Ca^2+^ free solution (*left)*. Cumulative events plotted versus time (*middle)* and the number of spikes per cell as mean ± SE in the presence (*n* = 16) or in the absence (*n* = 31) (*right*). Adapted from ref [14]. (d) Schematic view of α_1_1.2 subunit harboring the T1066Y mutation at IIIS6 that renders the channel Nif-resistant, and the L775P mutation at IIS6 that renders the channel Ca^2+^ impermeable. (e) Macroscopic whole-cell Ca^2+^ currents (*I*_Ca_) elicited from a holding potential of −80 mV to various test potentials in response to a 200 ms test pulse in *Xenopus* oocytes expressing GFP-tagged α_1_1.2 or GFP-tagged α_1_1.2^L775P^ with α2δ/β2A (*inset*). Representative traces and leak-subtracted peak current-voltage relationships of wt Cav1.2 and impermeable channel. Data collected from oocytes (*n* = 12 − 15), expressing α_1_1.2/α2δ/β2A (open circle), α_1_1.2^L775P^/α2δ/β2A (close circle), and α2δ/β2A subunits (rectangle). (f) Amperometry currents triggered by K60 from single bovine chromaffin cells infected with either Nif resistance wt α_1_1.2 or Nif resistance impermeable α_1_1.2^L775P^ mutant with 2 mM Ca^2+^ as the charge carrier, with or without Cav1.2 agonist FPL (0.5 μM) in the presence of 5 μM Nif. (g) Cumulative distribution of spikes plotted *versus* time after the onset of membrane depolarization in cells infected with wt α_1_1.2 subunit (*left panel*) or mutated α_1_1.2^L775P^ subunit (*right panel*) in the presence (●, ♦) and in the absence of 0.5 μm FPL (○, ◇), respectively. *Inset*, expanded scale of the cumulative events elicited by K60 from single cells infected with either wt α_1_1.2 subunit (*left panel*) or impermeable α_1_1.2/L775P (*right panel*) with 2 mM Ca^2+^ as the charge carrier, emphasizing initial rates (10–30 s) and sustained rates (30–60 s). Adapted from ref [63].

Amperometry experiments were performed in single bovine chromaffin cells to study catecholamine (CA) release mediated upon substituting Ca^2+^ ions with La^3+^ (Figure 6(a)). Membrane depolarization (60 mM KCl; 3 min) demonstrated that substitution of Ca^2+^ ions with La^3+^ triggered CA release. Evoked release during membrane depolarization was fully blocked in the presence of nifedipine (Nif), a selective Cav1.2 blocker, known to prevent conformational signaling by holding the channel in its inactive state (Figure 6). Hence, Cav1.2 ion-pore occupancy with either Ca^2+^ (Figure 6(b)) or La^3+^ (Figure 6(c)) appears to mediate depolarization-evoked CA release [14–16].

Non-ionotropic signaling was further examined by recording fusion pore kinetics, which provided insights into the initiation of the fusion process at the cell membrane [100]. In La^3+^ a shorter “foot”-width and a smaller “foot”-amplitude were revealed by an overall >50% reduction in “foot” charge [16]. Alterations in “foot” stability and “foot” size were observed also with Sr^2+^ and Ba^2+^ [15]. Alterations in fusion pore kinetics observed with different cations, indicate that the channel can regulate the fusion process, impacting both the size and stability of the fusion pore. This also implies that the channel serves as an integral structural component of the exocytotic release site [5–7,14,15].

The non-ionotropic initiation of vesicle fusion was additionally investigated using two L-type calcium channel-selective agonists, BayK8644 and FPL64176, which are known to enhance evoked release by increasing current frequency, current amplitude, and elevating cytosolic Ca^2+^. Depolarization evoked-release mediated in the presence of BayK-8644 or FPL64176 in La^3+^ allows for differentiation between the direct effect of the channel on the secretory machinery and subsequent effects resulting from intracellular ion elevation [63]. Single-cell recordings with La^3+^ as the charge carrier, revealed an acceleration in both the initial and the sustained rates of secretion triggered in the presence of BayK8644, and FPL64176. These changes, despite La^3+^ impermeability, imply that the enhanced release triggered by these agonists is independent of cation influx, indicating a direct functional interaction between the channel and secretory apparatus.

Differences in the kinetics of evoked-release shown to be affected mainly through the channel/Sx1A interaction were also found when Sr^2+^, or Ba^2+^ was used as a substitute for Ca^2+^ [15,16,63,101]. These results are in agreement with previous studies showing the ion-size dependence of the ability of the KcsA K^+^ channel to adopt a highly specific conductive structure, showing that ion-pore occupancy potentially influences distant site(s) within the channel [102]. These ion interactions (s) involve atoms at the selectivity filter and protein atoms surrounding the selectivity filter, extending up to a distance of 15 Å from the ions [102]; see also [103]

### Depolarization-triggered secretion mediated by Ca^2+^-impermeable Cav1.2 mutant

The non-ionotropic evoked-release exhibited by the La^3+^-bound ion pore was further validated through studies involving a single point Ca^2+^-impermeable channel mutant α_1_1.2^L775P^ [58] (Figure 6(d)). The ion-impermeability of this mutant and its targeting to the cell membrane were previously reported through patch clamp recordings, and fluorescence imaging in tsA-201 cells, and was later confirmed through two-electrode-voltage-clamp experiments conducted in *Xenopus* oocytes [13,58] (Figure 6(e)).

The mutated channel Cav1.2^L775P^, impermeable to Ca^2+^ or Ba^2+^, displayed compromised voltage-dependent monovalent conductance (*I*_Li+_), which was blocked by extracellular Ca^2+^. These properties indicate that the mutant preserves voltage sensitivity and maintains an intact Ca^2+^-binding site within the selectivity filter [13].

Amperometry recordings of the currents induced by this mutated channel were conducted in single bovine chromaffin cells, known to exclusively secrete catecholamines (CA) via nifedipine-sensitive Cav1.2 (as discussed earlier). The cells were infected with the Semliki Forest Virus, pSFV α_1_1.2^L775P/T1066Y^, a construct harboring the Ca^2+^-impermeable mutation L775P and the T1066Y mutation, which renders the channel nifedipine-resistant [13]. Membrane depolarization of cells infected with the Ca^2+^-impermeable mutant exhibited CA release in the presence of nifedipine (Figure 6(f)) [13,15,63]. The total CA release in the non-ionotropic mode, or total mean charge (TMC) values of the amperometric spikes calculated by the summation of spike area from each cell and averaged over the number of cells, was similar in cells expressing the Nif-insensitive α_1_1.2 subunit, the Nif-insensitive α_1_1.2^L775P/T1066Y^ subunit (in the presence of nifedipine), or in control GFP-infected cells (without nifedipine).

This non-ionotropic nature of release was further confirmed using the selective Cav1.2 agonist FPL64176 (see section above). Depolarization-evoked CA release in chromaffin cells expressing the impermeable Cav1.2^L775P/I1066Y^ or the wt Cav1.2 showed a comparable increase in the rate of secretion in the presence of FPL64176 (Figure 6(g)) [63]. These results imply that the enhanced rate of secretion is attributed to a conformational change induced by FPL64176 binding to the channel rather than Ca^2+^ influx (see channel agonist effects on CA release in La^3+^). These results are consistent with the structural alterations of the channel, affecting ES coupling independently of the elevation in [Ca^2+^]_i_. This supports the view that the channel operates as a signaling switch and is an integral part of the secretory machinery.

Notably, vesicle fusion driven by the non-ionotropic activity of the channel is critically dependent on extracellular Ca^2^**+**, which is essential for ion pore occupancy. This mechanism differs from the Ca^2+^-independent mechanism of vesicle fusion at the central synapse formed between the dorsal root ganglion and dorsal horn neurons [104].

#### The non-ionotropic activity of Cav1.2 mediates microsecond (µsec) evoked-release

The AZ typically consists of two distinct pools of synaptic vesicles (SV): i) the Readily-Releasable-Pool (RRP), comprised of readily-releasable, channel-associated Ca^2+^-primed vesicles, tightly associated with the membrane; and ii) the non-releasable or “tethered” pool of vesicles, situated farther from the membrane (5–10 nm), requiring Ca^2+^ for transition into the RRP state.

In the prevailing model, vesicle priming is achieved during Ca^2+^ binding to Syt1 C2 domains, forming a tight Syt1/SNARE/complexin macromolecular complex [105–108]. Accordingly, Syt1 serves as a Ca^2+^ sensor that triggers fast neurotransmitter release [109]. However, the interplay of Syt1/complexin/SNAREs is not fully understood, particularly how the “spring-loaded Syt1-SNARE-complexin” complex controls fast Ca^2+^-triggered fusion concomitantly with the prevention of premature fusion before Ca^2+^ influx occurs [76,109–111].

The insertion of Syt1 into the plasma membrane is imperative for vesicle docking. Facilitated by basic residues, this reaction is vital for engaging negatively charged phospholipids [112–116]. Similar to other C2 domain-containing proteins such as DOC2B, PLC, and PKC, the Ca^2+^-dependent membrane insertion in Syt1 takes place by Ca^2+^ binding to its C2A and C2B domains on a sub-second timescale. This suggests that vesicle priming by Ca^2+^ binding to Syt1 occurs within sub-second.

Moreover, given that Syt1 of a primed vesicle is Ca^2+^-bound (the priming step), it can no longer serve as a Ca^2+^ sensor for subsequent Ca^2+^-dependent vesicle fusion. Consequently, a Ca^2+^-dependent fusion of Ca^2+^-primed vesicles at the µsec timescale necessitates the involvement of a Ca^2+^-binding protein other than Syt1. A plausible candidate for this role is the Ca^2+^ channel, which not only binds Ca^2+^ but also colocalizes with the vesicle at the AZ and exhibits functional and physical interactions with Sx1A, SNAP-25, and Syt1.

Therefore, in agreement with the non-ionotropic activity of the Ca^2+^ channel, an alternative model was proposed, in which a conformational switch conveyed from the open channel through a transmembrane interplay with Sx1A/SNAP25 facilitates µs Ca^2+^-dependent neurotransmission. The binding of Ca^2+^ to the open ion pore confers Ca^2+^-dependency on this conformationally triggered vesicle fusion.

This model of transmitter release triggered by conformational changes, was further implied by a Total Internal Reflection fluorescence (TIRF) study in the cerebellar-mossy fiber terminal [78].

This study reported two distinct phases of vesicle fusion. During the first action potential (AP), vesicles tethered at release sites within a proximity of <100 nm from the AZ underwent a process lasting approximately 300–400 milliseconds (ms) without fusion. Subsequently, these vesicles that entered a state of readiness, were fused instantly by a second incoming AP [78]. These results showed an initial [Ca^2+^]-dependent docking/priming step of ~400 ms, potentially involving the binding of Ca^2+^ to Syt1 and subsequent rearrangement with complexin, Munc13, and SNAREs. The subsequent µseconds Ca^2+^-dependent step implies fusion of primed vesicles through conformational changes transmitted from Ca^2+^ bound VGCC to the exocytotic machinery.

Accordingly, Syt1/Munc13/complexin is implicated as the Ca^2+^ sensor(s) of vesicle priming (~400 ms), while the calcium channel is proposed to function as a putative Ca^2+^ sensor protein responsible for rapid/voltage-driven/conformationally triggered vesicle fusion [11,12].

### Spontaneous vesicle fusion explained by the non-ionotropic activity of the channel

The non-ionotropic activity of VGCC can potentially explain the spontaneous release of neurotransmitters from synaptic vesicles without the occurrence of an action potential. According to Fatt and Katz, spontaneous excitation “might simply be the result of excessive voltage noise across the nerve membrane … and may occasionally exceed the threshold level at some point. indicated random fluctuations of the resting potential due to thermal agitations within the membrane.” [117].

As a result, random fluctuations in the membrane potential coinciding with the stochastic opening of the Ca^2+^ channel may drive the fusion of channel-associated RRP. Thus, in addition to serving as a Ca^2+^ sensor in depolarization evoked release, the Ca^2+^ channel appears to be directly involved in triggering release in the absence of an action potential, or spontaneous release, which is a predominantly Ca^2+^-dependent process [118,119].

#### In summary

The alternative model of neurotransmitter release, distinct from the current model, incorporates Ca^2+^ channels, known to colocalize with the exocytotic machinery, providing the high Ca^2+^ concentrations required to trigger rapid vesicle fusion.

ES coupling necessitates the coordinated *ionotropic* and *non-ionotropic* activities of VGCCs. *Ionotropic* activity predominantly facilitates vesicle priming and signal termination, whereas *non-ionotropic* activity contributes to the conformation-induced vesicle fusion.

The brief microsecond interval between depolarization and vesicle fusion implies an actively functional Ca^2+^ channel complex with Ca^2+^-primed (RRP) proteins.

Mutational analysis and oxidation of the two highly conserved Sx1A TMD vicinal Cys residues revealed an intramembrane conformational-triggered signaling pathway between the exocytotic machinery and the channel. This pathway facilitates the propagation of depolarization-mediated conformational change(s), requiring Ca^2+^ binding to the open ion pore, thereby triggering high precision µs vesicle fusion through intramembrane channel/Sx1A signaling, preceding Ca^2+^-influx. Close proximity ensures a coordinated voltage and Ca^2+^ dependent vesicle fusion.

**Figure 7.**
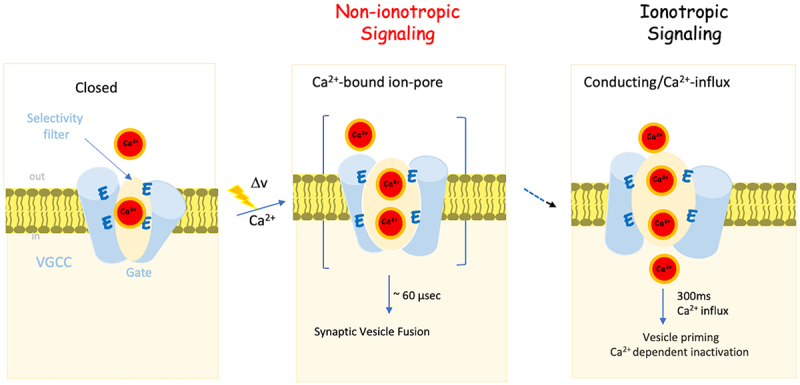
The non-ionotropic activity of Cav1.2 mediates excitation secretion (ES) coupling. A schematic illustration of the closed state of VGCCs in the closed state occupied by a single Ca^2+^ ion tightly bound (<1 µM) to the EEEE motif (*left)*. Upon arrival of an action potential (xxxv), the open channel, now occupied with additional Ca^2+^ ions, triggered fast (µs) fusion of a functionally VGCC-associate primed vesicle, prior to ion flow (non-ionotropic activity) (*middle*). Ca^2+^ inflow (ionotropic activity) elevates [Ca^2+^]_i_, which is essential for operating Ca^2+^-dependent intracellular activities, such as VGCC inactivation (CDI), and vesicle priming (*right*).

Unveiling this non-ionotropic/receptor-like signaling mechanism adds a novel dimension to our understanding of vesicle fusion. The non-ionotropic activity of the channel highlights its capacity to activate intracellular pathways not only through calcium influx but also by functioning as a Ca^2+^-binding macromolecule (Figure 7).

In the absence of an alternative plausible mechanism of microsecond Ca^2+^-triggered vesicle fusion, or an explanation for evoked release through Ca^2+^-impermeable channels or through channel-impermeable cations, the non-ionotropic induced synaptic transmission has emerged as a credible model of fast neurotransmission.

## Non-ionotropic excitation contraction (EC) coupling in cardiac cells

### Prevailing model calcium-induced-calcium-release(CICR)

EC coupling in skeletal muscle is initiated during the upstroke of a single action potential (AP) and proceeds independently of extracellular Ca^2+^. This process is facilitated by a physical association of the T-tubule membrane proximity to the sarcoplasmic reticulum between the L-type channel (Cav1.1) and ryanodine receptor 1 (RyR1) [120–123].

Unlike skeletal muscle, EC-coupling requires the presence of extracellular Ca^2+^ in cardiac cells. This requirement was demonstrated in rat cardiomyocytes that failed to trigger Ca^2+^ release from fully loaded junctional sarcoplasmic reticulum (jSR) stores without Ca^2+^ in the extracellular medium. In contrast to skeletal muscle, adult ventricular cardiomyocytes lack cross-junctional physical linkages between Cav1.2 and RyR2. However, clusters of Ca_V_1.2 on t-tubules have been known to be closely associated with the nanometer proximity of RyR2 clusters on jSR [124].

The prevailing model suggests that activating myofilaments and the contractile machinery proceeds by a small [Ca^2+^]_i_ rise (sparklet) upon Cav1.2 opening followed by a more substantial Ca^2+^ release from the SR via RyR2 (spark). This mechanism, termed Ca^2+^-induced Ca^2+^-release (CICR), has established Cav1.2 inward current (*I*_Ca_) as a mandatory requirement for cardiac contraction through the induction of Ca^2+^ mobilization from the intracellular stores of the SR [125,126]. Rapid application of small Ca^2+^-concentrations in skinned mammalian cardiac muscle strips caused larger release of cytosolic Ca^2+^, further supporting CICR as the mechanism of EC coupling in cardiac cells [127]. According to this CICR model, Ca^2+^ release from the SR also shuts off RyR2 opening and actively transports Ca^2+^ back into the SR, leading to muscle relaxation [128].

In the last decade, a significant number of additional proteins have been shown to enhance EC coupling efficiency in mammalian cardiomyocytes [129]. Cooperative gating of Ca_V_1.2 clusters in dyadic regions was suggested to reconcile a high probability of a spark with a relatively low probability of channel opening and a small amount of Ca^2+^ entry (see review [130]). More recently, the reproducibility of cardiac contraction has been attributed to the higher efficacy associated with high Ca^2+^ signaling variability at the subcellular, cellular, and network levels, produced by stochastic fluctuations in multiple processes in time and space [131]. An increase in the expression of newly inserted Cav1.2 and β adrenergic receptors in the t-tubule membrane has been shown to amplify Ca^2+^ influx, stimulating larger CICR and eliciting stronger contraction, thus overcoming the rather small Ca^2+^ influx in myocytes [132,133].

### Is Ca^2+^ entry mandatory for eliciting EC coupling? Issues related to the mechanism of cardiac contractility

Although the Ca^2±^induced Ca^2+^-release (CICR) mechanism is widely accepted as the dominant mechanism of cardiac contraction, several questions and challenges persist. Challenges include the absence of a distinct termination signal, unanticipated voltage sensitivity, and the absence of a well-defined Ca^2+^-binding protein targeted by *I*_ca_-gated Ca^2+^ as described below.

1. A critical issue involves the identification of a specific target protein for Ca^2+^ ions (sparklets). For a cellular response triggered by Ca^2+^ ions, similar to any other ligand, binding must occur at a selective and well-defined Ca^2+^ binding site. However, such a Ca^2+^ binding site, targeted by Ca^2+^ entry following Cav1.2 activation (sparklet), has not yet been conclusively identified.
2. A critical challenge arises from the incongruence between the low probability of Ca^2+^ entry and the requisite high probability of a spark. The relatively low probability of channel opening and the limited amount of Ca^2+^ entry (sparklet) through an individual Cav1.2 channel typically around 300-400 ions [134,135], is not consistent with the high probability of Ca^2+^ release from the SR (spark). An EF-hand domain found within RyR2, which is a high affinity Ca^2+^-binding site, is not required for RyR2 activation by cytosolic Ca^2+^ [136,137]. In a more recent study, a cooperative gating mechanism of Cav1.2 clusters in dyadic regions has been suggested to reconcile the high probability of a spark with a relatively low probability of channel opening [130]. Although cooperative interactions might provide a potential resolution, it is still somewhat controversial because it refutes the long-standing Hodgkin – Huxley hypothesis of ion channels as gating in a mutually independent manner. Cooperative gating is subject to regulatory control by key signaling pathways.
3. A critical issue with CICR is a steep voltage-dependency and insensitivity to intracellular Ca^2+^ CICR gain that displays a steep voltage-dependent activity between −30 to + 10 mVs and demonstrates resistance to intracellular Ca^2+^-buffering [138,139] (see also [140]). These observations suggest that Ca^2+^ reaches its target sites, spanning only nanometers away and acting before being captured by the buffers, or sequestered in a space inaccessible to them [141,142].

Lastly, 4) Another critical issue is studies showing cardiac contraction triggered by substituting Ca^2+^ with barium ions (Ba^2+^). Earlier studies have shown that cardiac muscle contraction can be triggered by replacing extracellular Ca^2+^ with Sr^2+^, or Ba^2+^ [143–145], see also [146]. Unlike Ca^2+^, Ba^2+^ fails to mediate cardiac muscle relaxation, mainly due to impaired Na^+^-Ba^2+^ exchange of the cardiac Na^+^/Ca^2+^ exchanger (NCX1), and poor substitution of Ca^2+^ in Cav1.2 calcium-dependent-inactivation (CDI) [147,148]. These results imply that a two-phase process in cardiac contractility is aligns with data demonstrating that more than 70% of the contractile force is initiated within the first 20–50 ms of the action potential (AP), and the remaining 300 ms appear to regulate the final stage of the contraction event [128,149].

One might speculate that the initial voltage-induced cardiac contraction occurs concurrently with Ca^2+^-occupancy of the low-affinity Cav1.2 EEEE motif and is supported by both Ca^2+^ and Ba^2+^. These data further imply that the slower step corresponds to the inactivation or termination phase, which is supported by Ca^2+^ binding to calmodulin. This support is absent for Ba^2+^ ions, as they do not bind to calmodulin. Hence, this model negates the EF hand as a target site, and requires further exploration to validate these intriguing observations.

As detailed above, open issues primarily resolve around whether Ca^2+^ entry is a mandatory requirement for triggering cardiac contractility. These challenges include the identification of a well-defined Ca^2+^ binding site targeted by Ca^2+^-sparklet, reconciliation of the low probability of Ca^2+^ entry with the high probability of a spark, understanding of a steep voltage-dependency, and insensitivity to Ca^2+^ buffering.

### Non-ionotropic activity of Cav1.2 serves as the on/off switch to mediate EC coupling in cardiomyocytes; Cav1.2 as the on/off switch of cardiac contraction

The unresolved mechanistic aspects of CICR, as mentioned above, have prompted an exploration of the non-ionotropic compatibility of Cav1.2, which triggers cardiac contraction.

The calculated distance of a 12–15-nm dyadic cleft between the junctional sarcoplasmic reticulum (jSR) and the plasma membrane implies a functional and physical association between clusters of Cav1.2 channels located on the t-tubules opposite to clusters of RyR2 on the jSR. This association is particularly evident when considering a 12 nm protrusion of the cytosolic portion of RyR2 into the cleft, while Cav1.2 is thought to protrude 2 nm into the same cleft.

The close proximity of Cav1.2 and RyR2 strengthens the likelihood of non-ionotropic conformational changes being transmitted to mediate cardiac contraction, as demonstrated in both ET and ES coupling.

The investigation of non-ionotropic-mediated EC coupling in neonatal cardiomyocytes further contributes to our understanding of the mechanism of cardiac contraction [21].

In this study, neonate cardiomyocytes were infected with a lentivirus encoded by a nifedipine (Nif)-resistant Ca^2+^-impermeable mutant α_1_1.2^L775P/T1066Y^. The Nif-resistance mutation was used to distinguish between endogenous channel signaling and the signaling of infected wt Cav1.2 or Ca^2+^-impermeable Ca1.2^L7745P/T1066Y^ channels [21].

Depolarization-induced cardiomyocyte contraction was assessed using the fluorescence ratio of the Ca^2+^-sensitive dye Indo-1 in neonatal cardiomyocytes expressing the Ca^2+^-impermeable mutant α_1_1.2^L775P/T1066Y^. The cells were preloaded with Indo-1 and depolarization was triggered electrically at 20–50 V for 10 ms with a frequency of 0.6 Hz, in the presence of Nif.

The Ca^2+^-impermeable channel mediated depolarization-triggered cardiomyocytes contraction is consistent with non-ionotropic and direct Cav1.2/RyR2 signaling.

**Figure 8.**
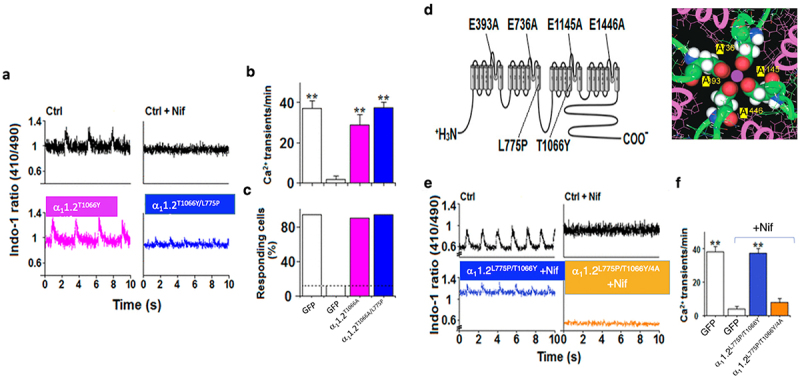
Ca^2+^-impermeable channel Cav1.2^L745P^ mediates excitation contraction (EC) coupling in cardiomyocytes, and requires intact selectivity filter. (a) Cardiac excitation – contraction coupling triggered by electrical stimulation in the absence (*left upper*) and presence (*right upper*) of 8 μM Nif in control intact cardiomyocytes, in cells infected with the Nif-resistant functional α_1_1.2^T1066Y^ subunit (*left lower*) and the Nif-resistant α_1_1.2^L775P/T1066Y^ mutant, in the presence of 8 μM Nif (*right lower*). (b) Frequency of Ca^2+^ transients in the presence and absence of Nif of control cells, and cells infected with α_1_1.2^T1066Y^ or α_1_1.2^L775P/T1066Y^. Data are shown as means ± SEM and analyzed by a Student’s *t* test. **p < 0.001 (*n* = 20). (c) Most of the cardiomyocytes infected with the Nif-resistant α_1_1.2^T1066Y^ (90%) or Nif-resistant α_1_1.2^L775P/T1066Y^ (94%), responded to cell stimulation in the presence of Nif, as compared with 12% of the control cells. (d), Schematic view of the α_1_1.2 subunit of Cav1.2 harboring the T1066Y mutation at IIIS6, providing Nif-resistant, a L745P mutation at IIS6, providing Ca^2+^-impermeability, and quadruple mutations EEEE/AAAA preventing ion-pore occupancy. (e) Representative 410 nm to 490 nm traces elicited in response to electric stimulation in control cells in the absence (*top, left*) and presence of 8 μM Nif (*top right*), α_1_1.2^L775P/T1066Y^ infected cells (*bottom left*), or α_1_1.2^L775P/T1066Y/4A^ infected cells (*bottom right*) in the presence of 8 μM Nif. (f) Frequency of depolarization-evoked Ca^2+^ transients in control and infected cells. Data is shown as means ± SEM and analyzed by a Student’s *t* test. **p < 0.001 (*n* = 20). Adapted from ref [21].

The amplitude of spontaneous Ca^2+^ transients was reduced by only 30% in α_1_1.2^T1066Y^-infected cells and by 50% in α_1_1.2^L775P/T1066Y^-infected cells, compared to the control GFP-infected cells in the absence of Nif. The voltage-activated Ca^2+^-impermeable channel α_1_1.2^L775P/T1066Y^ elicited Ca^2+^ transients at a frequency (37.3 ± 2.7 transients/min) similar to that of the α_1_1.2^T1066Y^ channel (28.8 ± 5.2 transients/min), or to GFP-infected cells (36.8 ± 3.8 transients/min) obtained in the absence of Nif (Figure 8(a-c)).

To ascertain that EC coupling requires extracellular Ca^2+^ for the occupancy of the ion pore and not inside the cell, the EEEE motif of the Ca^2+^-impermeable channel α_1_1.2^L775P/T1066Y^, which mediates cardiac contraction, was mutated to AAAA (Figure 8(d)). The resulting pore-mutant α_1_1.2^L775P/T1066Y/EEEE/AAAA^ elicited no contraction [21].

**Figure 9.**
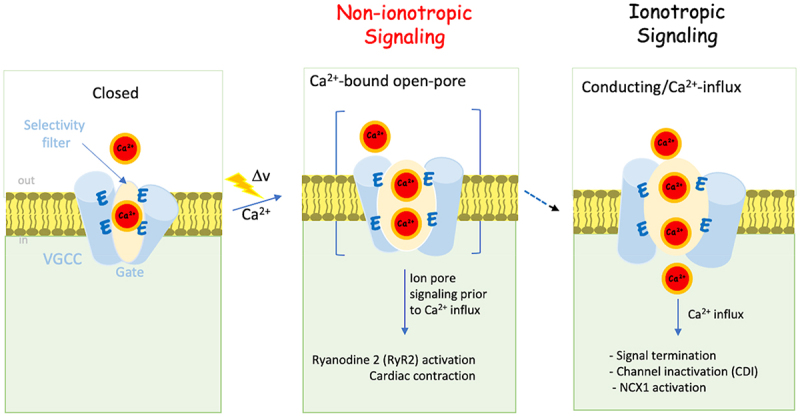
The non-ionotropic activity of Cav1.2 mediates excitation contraction (EC) coupling in cardiac cells. A schematic illustration of the closed state of VGCCs occupied by a single Ca^2+^ ion tightly bound (<1 µM) to the EEEE motif (*left)*. Upon arrival of an action potential (xxxv), the open channel, now occupied with an additional Ca^2+^ ion(s), triggers cardiac contraction most likely through a direct interaction with RyR2 (an unidentified site), prior to ion flow (non-ionotropic activity) (*middle*). Ca^2+^ inflow (ionotropic activity) elevates [Ca^2+^]_i_, which is essential for Ca^2+^-dependent intracellular activities, for example, replenishing SR stores, Cav1.2 inactivation (*right*).

These data imply that Cav1.2 functions as a calcium-binding protein, and mediation of cardiac contraction is critically dependent on ion-pore-occupancy rather than ion influx (Figure 8(e,f)). The results led to the proposal of a Ca^2+^-dependent high-fidelity model in which conformational changes at the channel are transduced from clusters of Cav1.2 directly to clusters of RyR2, prior to and independent of Ca^2+^ influx, similar to ET and ET coupling (see ET and ES coupling [13,23]) (Figure 9).

This non-ionotropic mechanism suggests that Cav1.2/RyR2 clusters can assemble to form functionally active complexes. This is consistent with the nanoscale functional organization of Cav1.2 clusters concentrated on t-tubules at dyadic junctions, which are assembled in close proximity (~12 nm) to RyR2 clustered on jSR [130,150–152]. Although the Cav1.2/RyR2 interface(s) remain unidentified, the prospect of a physical/functional link between the intracellular-domains of Cav1.2 and RyR2 May 2001 enable the direct transfer of conformationally triggered myocardial contraction.

Previous studies have shown functional and physical interactions between Cav1.3 and RyR2 [57]. In hippocampal CA1 neurons, fluctuations in the resting membrane potential appear to be sufficient to initiate functional coupling between the VGCC and RyR [153]. These studies suggest that juxtaposed localization of channels and RyRs offers an anatomical advantage, facilitating the synchronization of Ca^2+^ release from RyRs upon channel opening.

The non-ionotropic model of cardiac contraction is also consistent with the voltage-dependence of intracellular Ca^2+^ release, which mirrors a bell-shaped profile that corresponding to the voltage-dependence of Cav1.2 [146]. This bell-shaped *I*_Ca_-gated Ca^2+^ release mechanism aligns with the channel opening and ion-pore occupancy prior to cell entry, further supporting the proposed non-ionotropic model.

In the model where signaling by conformational change(s) is directly transferred to RyR2, the closure of Cav1.2 can also function as a termination signal. Accordingly, the kinetics of voltage-driven Cav1.2 opening/closure reverberate the kinetics of RyR2 opening/closure. This implies that the activation/inactivation of Cav1.2 May correspond to the on/off switch of Ca^2+^-dependent activation/inactivation of dyadic Ca^2+^ release.

The data obtained from rainbow trout heart experiments, as reported by Cros et al. [154], showed that, under control conditions, the initiation of cardiac contraction predominantly relies on extracellular Ca^2+^ rather than Ca^2+^ influx through Cav1.2 channels (I_Ca_). This finding lends further support to the non-ionotropic model of cardiac contraction.

#### In summary

While it is widely accepted that skeletal muscle contraction via Cav1.1/RyR1 remains independent of extracellular Ca^2+^, cardiac contractility through Cav1.2/RyR2 is dependent on extracellular Ca^2+^ and involves the Ca^2+^ influx-dependent CICR mechanism. Despite its well-established role, CICR, does not fully satisfy all the mechanistic features of excitation-triggered cardiac contractions.

The non-ionotropic mechanism of EC coupling proposes a conformational-coupling signaling process between Cav1.2 and RyR2 activated during the upstroke of an AP as the driver of cardiac contractility. Contraction is mediated by the non-canonical Cav1.2 activity, which depends on bell-shape *I*_Ca_ and ion-pore occupancy prior to Ca^2+^ influx, satisfying the mandatory presence of extracellular Ca^2+^. In this model Cav1.2 acts as a macromolecule transducing external stimuli to intracellular input rather than serving as a vehicle that elevates intracellular Ca^2+^.

Moreover, in the non-ionotropic model, certain issues become irrelevant, such as the necessity to identify a Ca^2+^ binding protein targeted by [Ca^2+^]_i_ rise (sparklet), or the increase in Ca^2+^ concentration at the channel mouth. This model aligns with observations of low synchronization of Ca^2+^ entry, steep voltage-dependency, insensitivity of CICR to Ca^2+^ buffering, and the ability of cardiac contraction to be mediated by barium ions (Ba^2+^).

The model predicts a reproducible and precisely coordinated process, favoring protein–protein interaction-based machinery with well-defined activation/termination signaling, as opposed to variability often associated with Ca^2+^ ion signaling.

Consistent with Cav1.2 non-ionotropic facilitating synaptic transmission, and gene activation, the non-ionotropic EC coupling in cardiac cells highlights the role of Cav1.2 as a transducer of external stimuli rather than merely a vehicle for elevating intracellular Ca^2+^.

While further investigations are warranted to confirm the precise regulation RyR2 by Cav1.2 opening and closure, *calcium-channel-induced-calcium-release* (CCICR) is posited to offer a more accurate description of the underlying mechanism of cardiac contraction, contrasting with the conventional-term calcium-*i*nduced-calcium-release (CICR).

Although additional studies are necessary to confirm the strict control of RyR2 by Cav1.2 opening and closure, calcium-channel-induced-calcium-release (CCICR) is proposed to better describe the actual mechanism of cardiac contraction, as opposed to calcium-induced-calcium-release (CICR).

*In conclusion*, the voltage gated calcium channel is a versatile molecule that is crucial for multiple physiological processes. While its recognized function involves calcium ion influx in response to membrane depolarization, the voltage gated calcium channel functions as a signaling macromolecule in a non-ionotropic mode. Remarkably, this non-canonical activity of the channel spans three systems: excitation-secretion (ES), excitation-contraction (EC), and excitation-transcription (ET), in which action potential-triggered conformational changes are coupled with subsequent Ca^2+^ occupancy of open ion-pores mediating rapid neurotransmitter release, gene activation, and cardiac contraction.

## Data Availability

Data sharing is not applicable to this article as no new data were created or analyzed in this study.

## References

[1] Gandini MA, Zamponi GW. Voltage-gated calcium channel nanodomains: molecular composition and function. FEBS J. 2022;289(3):614–27. doi: 10.1111/FEBS.15759 33576127

[2] Atlas D, Wiser O, Trus M. The voltage-gated Ca2+ channel is the Ca2+ sensor of fast neurotransmitter release. Cell Mol Neurobiol. 2001;21(6):717–731. doi: 10.1023/A:1015104105262 12043844 PMC11533850

[3] Ji J, Yang SN, Huang X, et al. Modulation of L-type Ca(2+) channels by distinct domains within SNAP-25. Diabetes. 2002;51(5):1425–1436. doi: 10.2337/DIABETES.51.5.1425 11978639

[4] Sheng ZH, Rettig J, Takahashi M. Identification of a syntaxin-binding site on N-type calcium channels. Neuron. 1994;13(6):1303–1313. doi: 10.1016/0896-6273(94)90417-0 7993624

[5] Wiser O, Bennett MK, Atlas D. Functional interaction of syntaxin and SNAP-25 with voltage-sensitive L- and N-type Ca2+ channels. Embo J. 1996;15(16):4100–4110. doi: 10.1002/j.1460-2075.1996.tb00785.x 8861939 PMC452132

[6] Wiser O, Tobi D, Trus M, et al. Synaptotagmin restores kinetic properties of a syntaxin-associated N-type voltage sensitive calcium channel. FEBS Lett. 1997;404(2–3):203–207. doi: 10.1016/S0014-5793(97)00130-0 9119064

[7] Wiser O, Trus M, Hernández A, et al. The voltage sensitive Lc-type Ca2+ channel is functionally coupled to the exocytotic machinery. Proc Natl Acad Sci U S A. 1999;96(1):248–253. doi: 10.1073/pnas.96.1.248 9874804 PMC15125

[8] Yang SN, Larsson O, Bränström R, et al. Syntaxin 1 interacts with the L D subtype of voltage-gated Ca 2+ channels in pancreatic β cells. Proc Natl Acad Sci U S A. 1999;96(18):10164–10169. doi: 10.1073/PNAS.96.18.10164 10468580 PMC17860

[9] Sabatini BL, Regehr WG. Timing of neurotransmission at fast synapses in the mammalian brain. Nature. 1996;384(6605):170–172. doi: 10.1038/384170a0 8906792

[10] Atlas D. Voltage-gated calcium channels function as Ca2±activated signaling receptors | Elsevier Enhanced Reader. Trends Biochem Sci. 2014;39(2):45–52. doi: 10.1016/j.tibs.2013.12.005 24388968

[11] Atlas D. Revisiting the molecular basis of synaptic transmission. Prog Neurobiol. 2022;216:102312. doi: 10.1016/J.PNEUROBIO.2022.102312 35760141

[12] Atlas D. The voltage-gated calcium channel functions as the molecular switch of synaptic transmission. Annu Rev Biochem. 2013;82(1):607–635. doi: 10.1146/annurev-biochem-080411-121438 23331239

[13] Hagalili Y, Bachnoff N, Atlas D. The voltage-gated Ca 2+ channel is the Ca 2+ sensor protein of secretion. Biochemistry. 2008;47(52):13822–13830. doi: 10.1021/bi801619f 19061337

[14] Marom M, Birnbaumer L, Atlas D. Membrane depolarization combined with Gq-activated G-protein-coupled receptors induce transient receptor potential channel 1 (TRPC1)- dependent potentiation of catecholamine release. Neuroscience. 2011;189. doi: 10.1016/j.neuroscience.2011.05.007 21621591

[15] Marom M, Sebag A, Atlas D. Cations residing at the selectivity filter of the voltage-gated Ca 2+ -channel modify fusion-pore kinetics. Channels. 2007;1(5):377–386. doi: 10.4161/chan.5398 18690038

[16] Lerner I, Trus M, Cohen R, et al. Ion interaction at the pore of Lc-type Ca2+ channel is sufficient to mediate depolarization-induced exocytosis. J Neurochem. 2006;97(1):116–127. doi: 10.1111/j.1471-4159.2006.03709.x 16515555

[17] Trus M, Corkey RF, Nesher R, et al. The L-type voltage-gated Ca 2+ channel is the Ca 2+ sensor protein of stimulus−secretion coupling in pancreatic beta cells. Biochemistry. 2007;46(50):14461–14467. doi: 10.1021/bi7016816 18027971

[18] Armstrong CM, Bezanilla FM, Horowicz P. Twitches in the presence of ethylene glycol bis(β-aminoethyl ether)-N,N′-tetraacetic acid. Biochim Biophys Acta. 1972;267(3):605–608. doi: 10.1016/0005-2728(72)90194-6 4537984

[19] Rios E, Brum G. Involvement of dihydropyridine receptors in excitation–contraction coupling in skeletal muscle. Nature. 1987;325(6106):717–720. doi: 10.1038/325717A0 2434854

[20] Tanabe T, Beam KG, Adams BA, et al. Regions of the skeletal muscle dihydropyridine receptor critical for excitation–contraction coupling. Nature. 1990;346(6284):567–569. doi: 10.1038/346567A0 2165570

[21] Gez LS, Hagalili Y, Shainberg A, et al. Voltage-driven Ca 2+ binding at the L-Type Ca 2+ channel triggers cardiac excitation–contraction coupling prior to Ca 2+ influx. Biochemistry. 2012;51(48):9658–9666. doi: 10.1021/bi301124a 23145875

[22] Servili E, Trus M, Atlas D. Ion occupancy of the channel pore is critical for triggering excitation-transcription (ET) coupling. Cell Calcium. 2019;84:102102. doi: 10.1016/J.CECA.2019.102102 31733625

[23] Servili E, Trus M, Maayan D, et al. β-Subunit of the voltage-gated Ca2+ channel Cav1.2 drives signaling to the nucleus via H-Ras. Proc Natl Acad Sci U S A. 2018;115(37):E8624–E8633. doi: 10.1073/pnas.1805380115 30150369 PMC6140482

[24] Servili E, Trus M, Sajman J, et al. Elevated basal transcription can underlie timothy channel association with autism related disorders. Prog Neurobiol. 2020;191:101820. doi: 10.1016/J.PNEUROBIO.2020.101820 32437834

[25] Krey JF, Paşca SP, Shcheglovitov A, et al. Timothy syndrome is associated with activity-dependent dendritic retraction in rodent and human neurons. Nat Neurosci. 2013;16(2):201–209. doi: 10.1038/NN.3307 23313911 PMC3568452

[26] Catterall WA, Lenaeus MJ, Gamal El-Din TM. Structure and pharmacology of voltage-gated sodium and calcium channels. Annu Rev Pharmacol Toxicol. 2020;60(1):133–154. doi: 10.1146/ANNUREV-PHARMTOX-010818-021757 31537174

[27] Dolphin AC. Functions of presynaptic voltage-gated calcium channels. Function. 2021;2(1):zqaa027. doi: 10.1093/function/zqaa027 33313507 PMC7709543

[28] Westhoff M, Dixon RE. Mechanisms and regulation of cardiac Cav1.2 trafficking. Int J Mol Sci. 2021;22(11):5927. doi: 10.3390/ijms22115927 34072954 PMC8197997

[29] Tang L, Gamal El-Din TM, Payandeh J, et al. Structural basis for Ca2+ selectivity of a voltage-gated calcium channel. Nature. 2014;505(7481):56–61. doi: 10.1038/NATURE12775 24270805 PMC3877713

[30] Chen Z, Mondal A, Abderemane-Ali F, et al. EMC chaperone–CaV structure reveals an ion channel assembly intermediate. Nature. 2023;619(7969):410–419. doi: 10.1038/S41586-023-06175-5 37196677 PMC10896479

[31] Sather WA, McCleskey EW. Permeation and selectivity in calcium channels. Annu Rev Physiol. 2003;65(1):133–159. doi: 10.1146/ANNUREV.PHYSIOL.65.092101.142345 12471162

[32] Bading H. Nuclear calcium signalling in the regulation of brain function. Nat Rev Neurosci. 2013;14(9):593–608. doi: 10.1038/NRN3531 23942469

[33] Bengtson CP, Bading H. Nuclear calcium signaling. Adv Exp Med Biol. 2012;970:377–405. doi: 10.1007/978-3-7091-0932-8_17 22351065

[34] Flavell SW, Greenberg ME. Signaling mechanisms linking neuronal activity to gene expression and plasticity of the nervous system. Annu Rev Neurosci. 2008;31(1):563–590. doi: 10.1146/ANNUREV.NEURO.31.060407.125631 18558867 PMC2728073

[35] Chrivia JC, Kwok RPS, Lamb N, et al. Phosphorylated CREB binds specifically to the nuclear protein CBP. Nature. 1993;365(6449):855–859. doi: 10.1038/365855A0 8413673

[36] Dolmetsch RE, Pajvani U, Fife K, et al. Signaling to the nucleus by an L-type calcium channel-calmodulin complex through the MAP kinase pathway. Science. 2001;294(5541):333–339. doi: 10.1126/SCIENCE.1063395 11598293

[37] Kornhauser JM, Cowan CW, Shaywitz AJ, et al. CREB transcriptional activity in neurons is regulated by multiple, calcium-specific phosphorylation events. Neuron. 2002;34(2):221–233. doi: 10.1016/S0896-6273(02)00655-4 11970864

[38] Stevenson AS, Cartin L, Wellman TL, et al. Membrane depolarization mediates phosphorylation and nuclear translocation of CREB in vascular smooth muscle cells. Exp Cell Res. 2001;263(1):118–130. doi: 10.1006/EXCR.2000.5107 11161711

[39] Wamhoff BR, Bowles DK, Owens GK. Excitation–transcription coupling in arterial smooth muscle. Circ Res. 2006;98(7):868–878. doi: 10.1161/01.RES.0000216596.73005.3C 16614312

[40] West AE, Chen WG, Dalva MB, et al. Calcium regulation of neuronal gene expression. Proc Natl Acad Sci U S A. 2001;98(20):11024–11031. doi: 10.1073/PNAS.191352298 11572963 PMC58677

[41] Wheeler DG, Barrett CF, Groth RD, et al. CaMKII locally encodes L-type channel activity to signal to nuclear CREB in excitation–transcription coupling. J Cell Bio. 2008;183(5):849–863. doi: 10.1083/JCB.200805048 19047462 PMC2592819

[42] Cohen SM, Li B, Tsien RW, et al. Evolutionary and functional perspectives on signaling from neuronal surface to nucleus. Biochem Biophys Res Commun. 2015;460(1):88–99. doi: 10.1016/J.BBRC.2015.02.146 25998737 PMC4701207

[43] Ma H, Groth RD, Cohen SM, et al. γCaMKII shuttles Ca^2+^/CaM to the nucleus to trigger CREB phosphorylation and gene expression. Cell. 2014;159(2):281–294. doi: 10.1016/J.CELL.2014.09.019 25303525 PMC4201038

[44] Cohen SM, Suutari B, He X, et al. Calmodulin shuttling mediates cytonuclear signaling to trigger experience-dependent transcription and memory. Nat Commun. 2018;9(1). doi: 10.1038/S41467-018-04705-8 PMC6015085 29934532

[45] Suzuki Y, Ozawa T, Kurata T, et al. A molecular complex of Ca v 1.2/CaMKK2/CaMK1a in caveolae is responsible for vascular remodeling via excitation–transcription coupling. Proc Natl Acad Sci USA. 2022;119(16). doi: 10.1073/PNAS.2117435119 PMC9169798 35412911

[46] Hagenston AM, Bading H, Bas-Orth C. Functional consequences of calcium-dependent synapse-to-nucleus communication: focus on transcription-dependent metabolic plasticity. Cold Spring Harb Perspect Biol. 2020;12(4). doi: 10.1101/CSHPERSPECT.A035287 PMC7111253 31570333

[47] Carter BC, Jahr CE. Postsynaptic, not presynaptic NMDA receptors are required for spike-timing-dependent LTD induction. Nat Neurosci. 2016;19(9):1218–1224. doi: 10.1038/NN.4343 27399842 PMC5003644

[48] Dore K, Malinow R. Elevated PSD-95 Blocks Ion-flux Independent LTD: A Potential New Role for PSD-95 in Synaptic Plasticity. Neuroscience. 2021;456:43–49. doi: 10.1016/J.NEUROSCIENCE.2020.02.020 32114099 PMC7483149

[49] Nabavi S, Kessels HW, Alfonso S, et al. Metabotropic NMDA receptor function is required for NMDA receptor-dependent long-term depression. Proc Natl Acad Sci U S A. 2013;110(10):4027–4032. doi: 10.1073/pnas.1219454110 23431133 PMC3593861

[50] Negri S, Faris P, Maniezzi C, et al. NMDA receptors elicit flux-independent intracellular Ca2+ signals via metabotropic glutamate receptors and flux-dependent nitric oxide release in human brain microvascular endothelial cells. Cell Calcium. 2021;99:102454. doi: 10.1016/J.CECA.2021.102454 34454368

[51] Park DK, Petshow S, Anisimova M, et al. Reduced d-serine levels drive enhanced non-ionotropic NMDA receptor signaling and destabilization of dendritic spines in a mouse model for studying schizophrenia. Neurobiol Dis. 2022a;170:170. doi: 10.1016/J.NBD.2022.105772 PMC9352378 35605760

[52] Park DK, Stein IS, Zito K. Ion flux-independent NMDA receptor signaling. Neuropharmacology. 2022b;210:109019. doi: 10.1016/J.NEUROPHARM.2022.109019 35278420 PMC9365119

[53] Rajani V, Sengar AS, Salter MW. Tripartite signalling by NMDA receptors. Mol Brain. 2020;13(1). doi: 10.1186/S13041-020-0563-Z PMC7029596 32070387

[54] Stein IS, Gray JA, Zito K. Non-Ionotropic NMDA receptor signaling drives activity-induced dendritic spine shrinkage. J Neurosci. 2015;35(35):12303–12308. doi: 10.1523/JNEUROSCI.4289-14.2015 26338340 PMC4556794

[55] Stein IS, Park DK, Flores JC, et al. Molecular Mechanisms of Non-ionotropic NMDA Receptor Signaling in Dendritic Spine Shrinkage. J Neurosci. 2020a;40(19):3741–3750. doi: 10.1523/JNEUROSCI.0046-20.2020 32321746 PMC7204083

[56] Trus M, Servili E, Taieb-Cohen T, et al. Autism associated mutations in β2 subunit of voltage-gated calcium channels constitutively activate gene expression. Cell Calcium. 2022;108:102672. doi: 10.1016/J.CECA.2022.102672 36427431

[57] Kim S, Yun HM, Baik JH, et al. Functional interaction of neuronal Cav1.3 L-type calcium channel with ryanodine receptor type 2 in the rat hippocampus. J Biol Chem. 2007;282(45):32877–32889. doi: 10.1074/JBC.M701418200 17823125

[58] Hohaus A, Beyl S, Kudrnac M, et al. Structural determinants of L-type channel activation in segment IIS6 revealed by a retinal disorder. J Biol Chem. 2005;280(46):38471–38477. doi: 10.1074/JBC.M507013200 16157588 PMC3189691

[59] Splawski I, Timothy KW, Sharpe LM, et al. Ca(V)1.2 calcium channel dysfunction causes a multisystem disorder including arrhythmia and autism. Cell. 2004;119(1):19–31. doi: 10.1016/J.CELL.2004.09.011 15454078

[60] Tran-Van-Minh A, De Waard M, Weiss N. Ca v β surface charged residues contribute to the regulation of neuronal calcium channels. Mol Brain. 2022;15(1). doi: 10.1186/S13041-021-00887-3 PMC8722133 34980202

[61] Splawski I, Timothy KW, Decher N, et al. Severe arrhythmia disorder caused by cardiac L-type calcium channel mutations. Proc Natl Acad Sci U S A. 2005;102(23):8089–8096. doi: 10.1073/PNAS.0502506102 15863612 PMC1149428

[62] Lipkind GM, Fozzard HA. Modeling of the outer vestibule and selectivity filter of the L-type Ca2+ channel. Biochemistry. 2001;40(23):6786–6794. doi: 10.1021/BI010269A 11389592

[63] Marom M, Hagalili Y, Sebag A, et al. Conformational changes induced in voltage-gated calcium channel Cav1.2 by BayK 8644 or FPL64176 modify the kinetics of secretion independently of Ca2+ influx. J Biol Chem. 2010;285(10):6996–7005. doi: 10.1074/jbc.M109.059865 20054004 PMC2844149

[64] Oliveria SF, Dittmer PJ, Youn DH, et al. Localized calcineurin confers Ca2±dependent inactivation on neuronal L-type Ca2+ channels. J Neurosci. 2012;32:15328–15337. doi: 10.1523/JNEUROSCI.2302-12.2012 23115171 PMC3593728

[65] Fowler T, Sen R, Roy AL. Regulation of primary response genes. Mol Cell. 2011;44(3):348–360. doi: 10.1016/J.MOLCEL.2011.09.014 22055182 PMC3212756

[66] Rienecker KDA, Poston RG, Segales JS, et al. Mild membrane depolarization in neurons induces immediate early gene transcription and acutely subdues responses to a successive stimulus. J Biol Chem. 2022;298(9):298. doi: 10.1016/J.JBC.2022.102278 PMC9396413 35863435

[67] Tyssowski KM, DeStefino NR, Cho JH, et al. Different neuronal activity patterns induce different gene expression programs. Neuron. 2018;98(3):530–546.e11. doi: 10.1016/J.NEURON.2018.04.001 29681534 PMC5934296

[68] Südhof TC. The synaptic vesicle cycle. Annu Rev Neurosci. 2004;27(1):509–547. doi: 10.1146/ANNUREV.NEURO.26.041002.131412 15217342

[69] Brunger AT, Choi UB, Lai Y, et al. Molecular mechanisms of fast neurotransmitter release. Annu Rev Biophys. 2018;47(1):469–497. doi: 10.1146/ANNUREV-BIOPHYS-070816-034117 29792815 PMC6378885

[70] Chapman ER. How does synaptotagmin trigger neurotransmitter release? Annu Rev Biochem. 2008;77(1):615–641. doi: 10.1146/ANNUREV.BIOCHEM.77.062005.101135 18275379

[71] Chapman ER. Synaptotagmin: A Ca2+ sensor that triggers exocytosis? Nat Rev Mol Cell Biol. 2002;3(7):498–508. doi: 10.1038/nrm855 12094216

[72] Park Y, Ryu JK. Models of synaptotagmin-1 to trigger Ca2+ -dependent vesicle fusion. FEBS Lett. 2018;592(21):3480–3492. doi: 10.1002/1873-3468.13193 30004579

[73] Rizo J. Mechanism of neurotransmitter release coming into focus. Protein Sci. 2018;27(8):1364–1391. doi: 10.1002/PRO.3445 29893445 PMC6153415

[74] Südhof T. Neurotransmitter release: The last millisecond in the life of a synaptic vesicle. Neuron. 2013;80(3):675–690. doi: 10.1016/j.neuron.2013.10.022 24183019 PMC3866025

[75] Südhof TC. A molecular machine for neurotransmitter release: synaptotagmin and beyond. Nat Med. 2013;19(10):1227–1231. doi: 10.1038/NM.3338 24100992

[76] Zhou Q, Zhou P, Wang AL, et al. The primed SNARE–complexin–synaptotagmin complex for neuronal exocytosis. Nature. 2017;548(7668):420–425. doi: 10.1038/NATURE23484 28813412 PMC5757840

[77] Brunger AT, Leitz J. The core complex of the Ca2±Triggered presynaptic fusion machinery. J Mol Biol. 2023;435(1):167853. doi: 10.1016/J.JMB.2022.167853 36243149 PMC10578080

[78] Miki T, Midorikawa M, Sakaba T. Direct imaging of rapid tethering of synaptic vesicles accompanying exocytosis at a fast central synapse. Proc Natl Acad Sci U S A. 2020;117(25):14493–14502. doi: 10.1073/pnas.2000265117 32513685 PMC7322003

[79] Bachnoff N, Cohen-Kutner M, Trus M, et al. Intra-membrane signaling between the voltage-gated Ca2±channel and cysteine residues of syntaxin 1A coordinates synchronous release. Sci Rep. 2013;3(1):1620. doi: 10.1038/srep01620 23567899 PMC3621091

[80] Barg S, Ma X, Eliasson L, et al. Fast exocytosis with few Ca2+ channels in insulin-secreting mouse pancreatic B cells. Biophys J. 2001;81(6):3308–3323. doi: 10.1016/S0006-3495(01)75964-4 11720994 PMC1301788

[81] Cohen R, Marom M, Atlas D, et al. Depolarization-evoked secretion requires two vicinal transmembrane cysteines of syntaxin 1A. PLOS ONE. 2007a;2(12):e1273. doi: 10.1371/JOURNAL.PONE.0001273 18060067 PMC2094736

[82] Tobi D, Wiser O, Trus M, et al. N-type voltage-sensitive calcium channel interacts with syntaxin, synaptotagmin and SNAP-25 in a multiprotein complex. Recept. 1998;6(2): 89–98. 9932286

[83] Trus M, Wiser O, Goodnough MC, et al. The transmembrane domain of syntaxin 1A negatively regulates voltage-sensitive Ca2+ channels. Neuroscience. 2001;104(2):599–607. doi: 10.1016/S0306-4522(01)00083-5 11377859

[84] Cohen-Kutner M, Nachmanni D, Atlas D. CaV2.1 (P/Q channel) interaction with synaptic proteins is essential for depolarization-evoked release. Channels (Austin). 2010;4. doi: 10.4161/CHAN.4.4.12130 20495360

[85] Cohen R, Atlas D. R-type voltage-gated Ca 2+ channel interacts with synaptic proteins and recruits synaptotagmin to the plasma membrane of Xenopus oocytes. Neuroscience. 2004;128(4):831–841. doi: 10.1016/j.neuroscience.2004.07.027 15464290

[86] Mochida S, Yokoyama CT, Kim DK, et al. Evidence for a voltage-dependent enhancement of neurotransmitter release mediated via the synaptic protein interaction site of N-type Ca2+ channels. Proc Natl Acad Sci U S A. 1998;95(24):14523–14528. doi: 10.1073/PNAS.95.24.14523 9826733 PMC24406

[87] Sajman J, Trus M, Atlas D, et al. The L-type voltage-gated calcium channel co-localizes with Syntaxin 1A in nano-clusters at the plasma membrane. Sci Rep. 2017;7(1):7. doi: 10.1038/S41598-017-10588-4 28900128 PMC5595989

[88] Cohen R, Schmitt BM, Atlas D. Molecular identification and reconstitution of depolarization-induced exocytosis monitored by membrane capacitance. Biophys J. 2005;89(6):4364–4373. doi: 10.1529/biophysj.105.064642 16150968 PMC1367000

[89] Arien H, Wiser O, Arkin IT, et al. Syntaxin 1A modulates the voltage-gated L-type calcium channel (Cavl.2) in a cooperative manner. J Biol Chem. 2003;278(31):29231–29239. doi: 10.1074/jbc.M301401200 12721298

[90] Cohen R, Marom M, Atlas D, et al. Depolarization-evoked secretion requires two vicinal transmembrane cysteines of syntaxin 1A. PLOS ONE. 2007b;2(12):e1273. doi: 10.1371/journal.pone.0001273 18060067 PMC2094736

[91] Schiavo G, Shone CC, Bennett MK, et al. Botulinum neurotoxin type C cleaves a single Lys-Ala bond within the carboxyl-terminal region of syntaxins. J Biol Chem. 1995;270(18):10566–10570. doi: 10.1074/JBC.270.18.10566 7737992

[92] Vardar G, Salazar-Lázaro A, Zobel S, et al. Syntaxin-1A modulates vesicle fusion in mammalian neurons via juxtamembrane domain dependent palmitoylation of its transmembrane domain. Elife. 2022;11. doi: 10.7554/ELIFE.78182 PMC9183232 35638903

[93] Cohen R, Schmitt BM, Atlas D. Reconstitution of depolarization and Ca2±evoked secretion in Xenopus oocytes monitored by membrane capacitance. Methods Mol Biol. 2008;440:269–282. doi: 10.1007/978-1-59745-178-9_21 18369953

[94] Bachnoff N, Trus M, Atlas D. Alleviation of oxidative stress by potent and selective thioredoxin-mimetic peptides. Free Radic Biol Med. 2011;50(10):1355–1367. doi: 10.1016/j.freeradbiomed.2011.02.026 21377525

[95] Dai XQ, Camunas-Soler J, Briant LJB, et al. Heterogenous impairment of α cell function in type 2 diabetes is linked to cell maturation state. Cell Metab. 2022;34(2):256–268.e5. doi: 10.1016/J.CMET.2021.12.021 35108513 PMC8852281

[96] Ramachandran S, Rodgriguez S, Potcoava M, et al. Single calcium channel nanodomains drive presynaptic calcium entry at lamprey reticulospinal presynaptic terminals. J Neurosci. 2022;JN-RM-2207–21. doi: 10.1523/jneurosci.2207-21.2022 PMC8944239 35063999

[97] Weiss N. Control of depolarization-evoked presynaptic neurotransmitter release by Ca v 2.1 calcium channel. Channels (Austin). 2010;4(6):431–433. doi: 10.4161/CHAN.4.6.13613 20935476 PMC3052244

[98] Aow J, Dore K, Malinow R. Conformational signaling required for synaptic plasticity by the NMDA receptor complex. Proc Natl Acad Sci U S A. 2015;112(47):14711–14716. doi: 10.1073/PNAS.1520029112 26553983 PMC4664326

[99] Dore K, Stein IS, Brock JA, et al. Unconventional NMDA receptor signaling. J Neurosci. 2017;37(45):10800–10807. doi: 10.1523/JNEUROSCI.1825-17.2017 29118208 PMC5678012

[100] Breckenridge LJ, Almers W. Currents through the fusion pore that forms during exocytosis of a secretory vesicle. Nature. 1987;328(6133):814–817. doi: 10.1038/328814A0 2442614

[101] Wiser O, Cohen R, Atlas D. Ionic dependence of Ca2+ channel modulation by syntaxin 1A. Proc Natl Acad Sci U S A. 2002;99(6):3968–3973. doi: 10.1073/pnas.052017299 11891287 PMC122632

[102] Lockless SW, Zhou M, MacKinnon R, et al. Structural and thermodynamic properties of selective ion binding in a K+ channel. PLoS Biol. 2007;5(5):1079–1088. doi: 10.1371/JOURNAL.PBIO.0050121 PMC1858713 17472437

[103] Brettmann JB, Urusova D, Tonelli M, et al. Role of protein dynamics in ion selectivity and allosteric coupling in the NaK channel. Proc Natl Acad Sci U S A. 2015;112(50):15366–15371. doi: 10.1073/PNAS.1515965112 26621745 PMC4687598

[104] Wang Y, Huang R, Chai Z, et al. Ca2+ -independent transmission at the central synapse formed between dorsal root ganglion and dorsal horn neurons. EMBO Rep. 2022;23(11). doi: 10.15252/EMBR.202154507 PMC9638852 36148511

[105] Giraudo CG, Eng WS, Melia TJ, et al. A clamping mechanism involved in SNARE-dependent exocytosis. Science. 2006;313(5787):676–680. doi: 10.1126/SCIENCE.1129450 16794037

[106] Melia TJ. Putting the clamps on membrane fusion: how complexin sets the stage for calcium-mediated exocytosis. FEBS Lett. 2007;581(11):2131–2139. doi: 10.1016/J.FEBSLET.2007.02.066 17350005

[107] Schaub JR, Lu X, Doneske B, et al. Hemifusion arrest by complexin is relieved by Ca2+–synaptotagmin I. Nat Struct Mol Biol. 2006;13(8):748–750. doi: 10.1038/NSMB1124 16845390

[108] Tang J, Maximov A, Shin OH, et al. A complexin/synaptotagmin 1 switch controls fast synaptic vesicle exocytosis. Cell. 2006;126(6):1175–1187. doi: 10.1016/J.CELL.2006.08.030 16990140

[109] Fernández-Chacón R, Königstorfer A, Gerber SH, et al. Synaptotagmin I functions as a calcium regulator of release probability. Nature. 2001;410(6824):41–49. doi: 10.1038/35065004 11242035

[110] Rizo J, Sari L, Qi Y, et al. All-atom molecular dynamics simulations of Synaptotagmin-SNARE-complexin complexes bridging a vesicle and a flat lipid bilayer. 2022. Elife 11. doi: 10.7554/ELIFE.76356 PMC9239685 35708237

[111] Voleti R, Jaczynska K, Rizo J. Ca2±dependent release of synaptotagmin-1 from the SNARE complex on phosphatidylinositol 4,5-bisphosphate-containing membranes. Elife. 2020;9:1–95. doi: 10.7554/ELIFE.57154 PMC7498268 32808925

[112] Chen Y, Wang YH, Zheng Y, et al. Synaptotagmin-1 interacts with PI(4,5)P2 to initiate synaptic vesicle docking in hippocampal neurons. Cell Rep. 2021;34(11):34. doi: 10.1016/J.CELREP.2021.108842 33730593

[113] Jaczynska K, Esquivies L, Pfuetzner RA, et al. Analysis of tripartite Synaptotagmin-1-SNARE-complexin-1 complexes in solution. FEBS Open Bio. 2023;13(1):26–50. doi: 10.1002/2211-5463.13503 PMC9811660 36305864

[114] Marín-Vicente C, Nicolás FE, Gómez-Fernández JC, et al. The PtdIns(4,5)P2 ligand itself influences the localization of PKCα in the plasma membrane of intact living cells. J Mol Biol. 2008;377(4):1038–1052. doi: 10.1016/J.JMB.2007.12.011 18304574

[115] Michaeli L, Gottfried I, Bykhovskaia M, et al. Phosphatidylinositol (4, 5)-bisphosphate targets double C2 domain protein B to the plasma membrane. Traffic. 2017;18(12):825–839. doi: 10.1111/TRA.12528 28941037 PMC5967617

[116] Sato M, Mori Y, Matsui T, et al. Role of the polybasic sequence in the Doc2α C2B domain in dense-core vesicle exocytosis in PC12 cells. J Neurochem. 2010;114(1):171–181. doi: 10.1111/J.1471-4159.2010.06739.X 20403080

[117] FATT F, KATZ K. Membrane potentials at the motor end-plate. J Physiol. 1950;111(1–2):46p–47p. 14795474

[118] Schneggenburger R, Rosenmund C. Molecular mechanisms governing Ca(2+) regulation of evoked and spontaneous release. Nat Neurosci. 2015;18(7):935–941. doi: 10.1038/NN.4044 26108721

[119] Xu J, Pang ZP, Shin OH, et al. Synaptotagmin-1 functions as a Ca2+ sensor for spontaneous release. Nat Neurosci. 2009;12(6):759–766. doi: 10.1038/nn.2320 19412166 PMC2739891

[120] Flucher BE, Campiglio M. STAC proteins: The missing link in skeletal muscle EC coupling and new regulators of calcium channel function. Biochim Biophys Acta, Mol Cell Res. 2019;1866(7):1101–1110. doi: 10.1016/J.BBAMCR.2018.12.004 30543836

[121] Franzini-Armstrong C. The relationship between form and function throughout the history of excitation–contraction coupling. J Gen Physiol. 2018;150(2):189–210. doi: 10.1085/JGP.201711889 29317466 PMC5806676

[122] Ríos E, Pizarro G. Voltage sensor of excitation-contraction coupling in skeletal muscle. Physiol Rev. 1991;71(3):849–908. doi: 10.1152/PHYSREV.1991.71.3.849 2057528

[123] Rufenach B, Van Petegem F. Structure and function of STAC proteins: Calcium channel modulators and critical components of muscle excitation–contraction coupling. J Biol Chem. 2021;297(1):297. doi: 10.1016/J.JBC.2021.100874 PMC8258685 34129875

[124] Kawai M, Hussain M, Orchard CH. Excitation-contraction coupling in rat ventricular myocytes after formamide-induced detubulation. Am J Physiol. 1999;277(2):H603–H609. doi: 10.1152/AJPHEART.1999.277.2.H603 10444485

[125] Fabiato A. Calcium-induced release of calcium from the cardiac sarcoplasmic reticulum. Am J Physiol. 1983;245(1):C1–C14. doi: 10.1152/AJPCELL.1983.245.1.C1 6346892

[126] Sham JSK, Cleemann L, Morad M. Functional coupling of Ca2+ channels and ryanodine receptors in cardiac myocytes. Proc Natl Acad Sci U S A. 1995;92(1):121–125. doi: 10.1073/PNAS.92.1.121 7816800 PMC42829

[127] Fabiato A. Time and calcium dependence of activation and inactivation of calcium-induced release of calcium from the sarcoplasmic reticulum of a skinned canine cardiac Purkinje cell. J Gen Physiol. 1985;85(2):247–289. doi: 10.1085/JGP.85.2.247 2580043 PMC2215800

[128] Bers DM. Cardiac excitation–contraction coupling. Nature. 2002;415(6868):198–205. doi: 10.1038/415198A 11805843

[129] Dixon RE, Trimmer JS. Endoplasmic reticulum-plasma membrane junctions as sites of depolarization-induced Ca2+ signaling in excitable cells. Annu Rev Physiol. 2023;85:217–243. doi: 10.1146/ANNUREV-PHYSIOL-032122-104610 36202100 PMC9918718

[130] Dixon RE, Navedo MF, Binder MD, et al. Mechanisms and physiological implications of cooperative gating of clustered ion channels. Physiol Rev. 2022;102(3):1159–1210. doi: 10.1152/PHYSREV.00022.2021 34927454 PMC8934683

[131] Guarina L, Moghbel AN, Pourhosseinzadeh MS, et al. Biological noise is a key determinant of the reproducibility and adaptability of cardiac pacemaking and EC coupling. J Gen Physiol. 2022;154(9). doi: 10.1085/JGP.202012613 PMC9059386 35482009

[132] Del Villar SG, Voelker TL, Westhoff M, et al. β-Adrenergic control of sarcolemmal CaV1.2 abundance by small GTPase Rab proteins. Proc Natl Acad Sci, USA. 2021;118(7):S. A. 118. doi: 10.1073/pnas.2017937118 PMC7896340 33558236

[133] Ito DW, Hannigan KI, Ghosh D, et al. β-adrenergic-mediated dynamic augmentation of sarcolemmal CaV 1.2 clustering and co-operativity in ventricular myocytes. J Physiol. 2019;597(8):2139–2162. doi: 10.1113/JP277283 30714156 PMC6462464

[134] Josephson IR, Guia A, Sobie EA, et al. Physiologic gating properties of unitary cardiac L-type Ca2+ channels. Biochem Biophys Res Commun. 2010;396(3):763–766. doi: 10.1016/J.BBRC.2010.05.016 20457123 PMC2892242

[135] Wang SQ, Song LS, Lakatta EG, et al. Ca2+ signalling between single L-type Ca2+ channels and ryanodine receptors in heart cells. Nature. 2001;410(6828):592–596. doi: 10.1038/35069083 11279498

[136] Guo W, Sun B, Xiao Z, et al. The EF-hand Ca2+ binding domain is not required for cytosolic Ca2+ activation of the cardiac ryanodine receptor. J Biol Chem. 2016;291(5):2150–2160. doi: 10.1074/JBC.M115.693325 26663082 PMC4732201

[137] Meissner G. The structural basis of ryanodine receptor ion channel function. J Gen Physiol. 2017;149(12):1065–1089. doi: 10.1085/JGP.201711878 29122978 PMC5715910

[138] Adachi-Akahane S, Cleemann L, Morad M. BAY K 8644 modifies Ca2+ cross signaling between DHP and ryanodine receptors in rat ventricular myocytes. American Journal Of Physiology-Heart And Circulatory Physiology. 1999;276(4):H1178–H1189. doi: 10.1152/AJPHEART.1999.276.4.H1178 10199841

[139] Altamirano J, Bers DM. Voltage dependence of cardiac excitation–contraction coupling. Circ Res. 2007;101(6):590–597. doi: 10.1161/CIRCRESAHA.107.152322 17641229

[140] Zhang XH, Morad M. Ca2+ signaling of human pluripotent stem cells-derived cardiomyocytes as compared to adult mammalian cardiomyocytes. Cell Calcium. 2020;90:102244. doi: 10.1016/J.CECA.2020.102244 32585508 PMC7483365

[141] Ríos E, Stern MD. Calcium in close quarters: microdomain feedback in excitation-contraction coupling and other cell biological phenomena. Annu Rev Biophys Biomol Struct. 1997;26(1):47–82. doi: 10.1146/ANNUREV.BIOPHYS.26.1.47 9241413

[142] Stern MD. Buffering of calcium in the vicinity of a channel pore. Cell Calcium. 1992;13(3):183–192. doi: 10.1016/0143-4160(92)90046-U 1315621

[143] Judd RM, Levy BI. Effects of barium-induced cardiac contraction on large- and small-vessel intramyocardial blood volume. Circ Res. 1991;68(1):217–225. doi: 10.1161/01.RES.68.1.217 1984864

[144] Saeki Y, Shibata T, Shiozawa K. Excitation-contraction coupling in mammalian cardiac muscle during Ba2±induced contracture. Am J Physiol. 1981;240(2):H216–H221. doi: 10.1152/AJPHEART.1981.240.2.H216 7468817

[145] Tibbits GF, Philipson KD. Na±dependent alkaline earth metal uptake in cardiac sarcolemmal vesicles. Biochim Biophys Acta. 1985;817(2):327–332. doi: 10.1016/0005-2736(85)90035-5 4016109

[146] Näbauer M, Callewaert G, Cleemann L, et al. Regulation of calcium release is gated by calcium current, not gating charge, in cardiac myocytes. Science. 1989;244(4906):800–803. doi: 10.1126/SCIENCE.2543067 2543067

[147] Kirchhefer U, Neumann J, Bers DM, et al. Impaired relaxation in transgenic mice overexpressing junctin. Cardiovasc Res. 2003;59(2):369–379. doi: 10.1016/S0008-6363(03)00432-2 12909320

[148] Trac M, Dyck C, Hnatowich M, et al. Transport and regulation of the cardiac Na(+)-Ca2+ exchanger, NCX1. Comparison between Ca2+ and Ba2+. J Gen Physiol. 1997;109(3):361–369. doi: 10.1085/JGP.109.3.361 9089442 PMC2217077

[149] Morad M, Trautwein W. The effect of the duration of the action potential on contraction in the mammalian heart muscle. Pflugers Arch Gesamte Physiol Menschen Tiere. 1968;299(1):66–82. doi: 10.1007/BF00362542 5243673

[150] Mitchell MR, Powell T, Terrar DA, et al. Electrical activity and contraction in cells isolated from rat and guinea-pig ventricular muscle: a comparative study. J Physiol. 1987;391(1):527–544. doi: 10.1113/JPHYSIOL.1987.SP016754 2451011 PMC1192230

[151] Scriven DRL, Dan P, Moore EDW. Distribution of proteins implicated in excitation-contraction coupling in rat ventricular myocytes. Biophys J. 2000;79(5):2682–2691. doi: 10.1016/S0006-3495(00)76506-4 11053140 PMC1301148

[152] Sun XH, Protasi F, Takahashi M, et al. Molecular architecture of membranes involved in excitation-contraction coupling of cardiac muscle. J Cell Bio. 1995;129(3):659–671. doi: 10.1083/JCB.129.3.659 7730402 PMC2120446

[153] Berrout J, Isokawa M. Homeostatic and stimulus-induced coupling of the L-type Ca2+ channel to the ryanodine receptor in the hippocampal neuron in slices. Cell Calcium. 2009;46(1):30–38. doi: 10.1016/J.CECA.2009.03.018 19411104 PMC2703683

[154] Cros C, Sallé L, Warren DE, et al. The calcium stored in the sarcoplasmic reticulum acts as a safety mechanism in rainbow trout heart. Am J Physiol Regul Integr Comp Physiol. 2014;307(12):R1493–R1501. doi: 10.1152/AJPREGU.00127.2014 25377479 PMC4269670

